# Proteome-transcriptome alignment of molecular portraits achieved by self-contained gene set analysis: Consensus colon cancer subtypes case study

**DOI:** 10.1371/journal.pone.0221444

**Published:** 2019-08-22

**Authors:** Galina Glazko, Boris Zybailov, Frank Emmert-Streib, Ancha Baranova, Yasir Rahmatallah

**Affiliations:** 1 Department of Biomedical Informatics, University of Arkansas for Medical Sciences, Little Rock, AR, United States of America; 2 Department of Biochemistry and Molecular Biology, University of Arkansas for Medical Sciences, Little Rock, AR, United States of America; 3 Computational Medicine and Statistical Learning Laboratory, Tampere University of Technology, Korkeakoulunkatu, Tampere, Finland FI; 4 School of Systems Biology, George Mason University, Manassas VA, United States of America; 5 Research Center for Medical Genetics, Moscow, Russia; National Center for Biotechnology Information, UNITED STATES

## Abstract

Gene set analysis (GSA) has become the common methodology for analyzing transcriptomics data. However, self-contained GSA techniques are rarely, if ever, used for proteomics data analysis. Here we present a self-contained proteome level GSA of four consensus molecular subtypes (CMSs) previously established by transcriptome dissection of colon carcinoma specimens. Despite notable difference in structure of proteomics and transcriptomics data, many pathway-wide characteristic features of CMSs found at the mRNA level were reproduced at the protein level. In particular, CMS1 features show heavy involvement of immune system as well as the pathways related to mismatch repair, DNA replication and functioning of proteasome, while CMS4 tumors upregulate complement pathway and proteins participating in epithelial-to-mesenchymal transition (EMT). In addition, protein level GSA yielded a set of novel observations visible at the proteome, but not at the transcriptome level, including possible involvement of major histocompatibility complex II (MHC-II) antigens in the known immunogenicity of CMS1 and a connection between cholesterol trafficking and the regulation of Integrin-linked kinase (ILK) in CMS3. Overall, this study proves utility of self-contained GSA approaches as a critical tool for analyzing proteomics data in general and dissecting protein-level molecular portraits of human tumors in particular.

## Introduction

The molecular profiles obtained in large scale omics experiments (most frequently gene expressions, protein abundances or metabolites) are far from being self-explanatory and comprehensive. They do not offer immediate insight into the difference between phenotypes or mechanism of disease. These profiles require further analysis and interpretation, and to understand the underlying biological processes behind phenotypic differences, the data typically are integrated with pre-existing biological knowledge, including e.g. biological pathways, protein-protein and drug-protein interaction data, disease-specific databases and other relevant information. Here we consider the very first and most popular integration step of molecular profiles with biological pathways, in a context of proteomics data.

Approaches that incorporate existing biological knowledge, in a form of functionally related gene sets or known biological pathways, into the analysis became a routine for transcriptomics data more than a decade ago (see [1] for a review). The simplest technique incorporating biological knowledge, designed toward interpreting long gene lists, is the gene set overrepresentation analysis. When two phenotypes are compared, a set of *a priori* selected, significantly differentially expressed (DE) genes, is tested for overrepresentation in annotated gene sets such as Gene Ontology (GO) categories or Kyoto Encyclopedia of genes and genomes (KEGG), or Molecular Signature Database (MSigDB), or any other pathway database using standard statistical tests for enrichment [2]. This particular technique is also widely used for the analysis of proteomics data—in a very straightforward manner. For proteomics data, instead of significantly DE genes, significantly differentially abundant (DA) proteins are selected and the same enrichment tests are utilized. The major obstacle in applying overrepresentation analysis for proteomics data is the same as for transcriptomics data: the procedure requires a list of entities, significantly different between two phenotypes. More often than not, due to the limited sample size, small changes in expression/abundance, large variance or other shortcomings, no significant calls for either genes or proteins could be made. In a review illustrating the application of overrepresentation analysis for proteomics data [3], the authors analyzed data set with *n*_*1*_ = 72 patients with Parkinson disease and *n*_*2*_ = 72 healthy controls [4]. Despite the sample size, which was rather large for proteomics experiment, after correction for multiple testing there were no DA proteins between two groups, and to perform exemplarily overrepresentation analysis unadjusted *p*-values were used, leading to high risk of false positives.

Our aim here is to present, in the context of proteomics data, an alternative technique that integrates proteomics data with biological pathways (*gene sets*) with no requirement for proteins to be selected *a priori*. These methodologies were developed for transcriptomics data and are collectively called Gene Set Analysis (GSA) approaches. In GSA, a gene set is treated as a unit of expression [5–8]; in proteomics GSA, a set of proteins is treated as a unit of abundance. GSA approaches are readily distinguished based on the null hypotheses they test and can be either *self-contained* or *competitive* [9]. *Self-contained* approaches compare whether a gene set is differentially expressed between two phenotypes, while *competitive* approaches compare a gene set against its complement that contains all genes except genes in the set [9,10]. A number of review articles concerning the different aspects of GSA approaches developed for transcriptomics data analysis has been published [6,9,11–16]. In the context of proteomics data, competitive GSA approaches are gradually becoming as popular as in the context of transcriptomics data (see [17,18] for a review). Even more, several competitive GSA tests were developed specifically for comprehensive analysis of proteomics data. These examples includes protein set enrichment analysis (PSEA) for protein set enrichment analysis (a competitive GSA approach similar to GSEA) [19] and PSEA-Quant, a version of protein set enrichment analysis allowing to analyze samples from single or multiple conditions [20] (competitive GSA approach similar to single sample extension of GSEA, ssGSEA [21]). However, to the best of our knowledge, self-contained approaches were rarely, if ever, applied to proteomics data, despite it was repeatedly shown that they have more power and lower Type I error rate, as compared to competitive tests [1,8,22]. To bridge this gap, here we demonstrate utility of *self-contained* GSA approaches in analyzing consensus colon cancer subtypes on proteomics data. For the sake of comparison we also present the results of competitive GSA tests, developed for transcriptomics data, for the same proteomics data set.

Colorectal cancer (CRC) is a heterogeneous disease with distinct molecular properties resulting in different clinical outcomes and 5-year survival below 60% [23]. Large variation in clinical outcomes emphasize the need to develop early detection and predictive biomarkers that are easily translated into clinical practice [24]. Initial clinical characterization of colon cancer is defined by its **TNM** (Tumor, Nodes, Metastasis) stage [25], a notation system that describes the stage of a cancer which originates from a solid tumor. **T** describes the size of the original (primary) tumor and whether it has invaded nearby tissue, **N** describes nearby lymph nodes that are involved, and **M** describes distant metastasis [26]. Despite its wide adoption, in many cases this staging system fails to provide a prognostic value or a guidance for treatment decisions, for example, this is true for stage II and III of CRC [25,27]. Gradually it became clear that CRC of different molecular phenotypes respond to the treatment differently. Now at least three major adenoma to carcinoma progression subtypes are well recognized: microsatellites instability (MSI) [28], chromosomal instability (CIN) [29] and the CpG Island Methylator Phenotype (CIMP) [30].

In the last two decades whole transcriptome analysis became routinely used to dissect cancer molecular subtypes correlating with clinical outcomes. Starting with the seminal paper of Golub et al [31], defining finer subclasses of the leukemias, there has been a steady growth in similarly designed research [32,33]. For colorectal cancer (CRC), six different transcriptome-based subtype classifications have been suggested by independent laboratories [34–39]. These classifications were seemingly distinct, with different number (3 to 6) of CRC subtypes and different molecular descriptors. This led to the establishment of the CRC Subtyping Consortium (CRCSC) in 2014, which aimed to refine CRC classification subtypes and find out potential overlaps between six published transcriptome-based classifications [40]. The CRCSC involved six participating groups that established six CRC classification system and an ‘evaluation group’ (Sage Bionetwork) that provided a platform for data sharing and analysis [40,41]. Finally CRCSC suggested a subtype identification framework more general than, for example, simple application of unsupervised clustering approaches for subtype identification. The CRCSC (1) re-classified merged datasets compiled from the data produced by all groups providing the original algorithms, (2) calculated a similarity matrix based on Jaccard coefficients between all subtypes (3) retained only subtypes with statistically significant associations, (4) formed a network of subtypes and (5) used Markov Cluster algorithm to split the network into four molecular subgroups named “Consensus Molecular Subtypes” (CMS) [40,41]. These include CMS1, defined by high mutation rate, encompassing most microsatellite instable (MSI) tumors with inactivating alternations in mismatch repair (MMR) genes. CMS1 was also characterized by increased expression of genes associated with diffuse immune infiltrate, in particular CD8^+^ cytotoxic T lymphocytes (CTL), CD4^+^ T helper (T_H_1) cells and natural killer (NK) cells (MSI immune) [40]. CMS2-CMS4 subtypes displayed higher chromosomal instability (CIN), with CMS2 characterized by epithelial differentiation and strong upregulation of WNT and MYC, CMS3 enriched in metabolic signatures and CMS4 defined as ‘mesenchymal-like’, with upregulation of genes involved in epithelial-to-mesenchymal transition (EMT) (see [40,41] for more detailed description of CMSs molecular properties).

As proteins link genotype to phenotype, for more detailed characterization of CRC subtypes, respective proteomes were also analyzed [42]. Using already available transcriptomes for the same samples, the authors found that protein abundance and gene expression level correlated only modestly, with about one third of correlations being statistically significant [42,43]. The five proteomic CRC subtypes A-E, identified using consensus clustering [42], were linked with genomic and epigenomic features (MSI, CIMP and CIN described above) only, since at that time (2014) CMS classification was not yet available, being presented a year later, in 2015 paper [40]. Yet, the authors of the 2015 consensus transcriptome-based subtypes classification [40] did compare four CMS groups with the five proteomic CRC subtypes and an approximate mapping between two classifications was observed (Supplementary Table 10 in the original study [40]). The authors also implemented gene set enrichment analysis with competitive GSA test [44] and found some similarities between transcriptome-based and proteome-based subtypes, in particular, CMS1 and CMS4, but no new pathways were found for proteomics data [40]. Here, we re-analyze previously published proteomes of CRC to elucidate to what extent transcriptionally identified CMS subtypes are detectable at the proteome level with self-contained GSA tests and if new pathways can be detected with self-contained tests.

## Methods

### Self-contained GSA tests

#### KS and RKS

The multivariate generalization of Kolmogorov-Smirnov (KS) statistic tests the null hypothesis of mean vectors equality between two phenotypes, while ‘radial’ Kolmogorov-Smirnov (RKS) statistic tests the variance vectors equality between two phenotypes and is sensitive to alternatives having similar mean vectors but differences in scale [45,46]. KS and RKS tests were used as implemented in Bioconductor package GSAR [46].

#### ROAST

Rotation gene set tests (ROAST) [47] uses the framework of linear models and tests whether for all genes in a set a particular contrast of the coefficients is non-zero [47]. It can account for correlations between genes and has the flexibility of using different alternative hypotheses, testing whether the direction of changes for a gene in a set is *up*, *down* or *mixed* (up or down) [47]. Instead of permutation, it uses rotation, a parametric resampling method suitable for linear models and therefore can have better p-values for rather small sample size [47]. ROAST test was used as implemented in limma Bioconductor package.

#### GSNCA

The Gene Sets Net Correlations Analysis (GSNCA) method detects the differences in net correlation structure for a gene set between two conditions [48] and was used as implemented in function GSNCAtest from Bioconductor package GSAR [46].

We applied KS, ROAST, RKS and GSNCA tests to find (1) differential expression (DE) (2) differential variability (DV) and (3) differential co-expression (DC) of gene sets between subtypes. The results of ROAST and KS tests were similar, and only ROAST results were included. RKS test did not find any statistically significant pathways and its results were not included.

In order to be included in the list of DE or DV pathways, a pathway was required to have a Benjamini-Hochberg adjusted *p*_*adj*_<0.01 after correction for multiple testing. Specifically for ROAST test a pathway was also required to include 1) more than 60% of up-regulated or more than 60% of down-regulated proteins and 2) at least 50% of the original pathway members. In order to be included in a list of DC pathways, a pathway was required to have an adjusted *p*_*adj*_<0.1 after correction for multiple testing.

### Competitive GSA tests

#### GSEA

The first competitive GSA test for microarray data analysis was Gene Set Enrichment Analysis (GSEA) method [49,50]. As a local test statistic it uses a signal to noise ratio and a weighted Kolmogorov-Smirnov as a global test statistic (enrichment score, normalized to factor out the gene set size dependence) [13,50]. GSEA tests the null hypothesis that the genes in a gene set are randomly associated with the phenotype. GSEA test was used as implemented on MSigDB GSEA home website (http://software.broadinstitute.org/gsea/index.jsp).

#### ROMER

Rotation testing using MEan Ranks (ROMER) tests the same hypothesis as GSEA, the only difference is that, similar to ROAST, instead of permutations it uses rotations to obtain p-values [51]. ROMER test was used as implemented in limma Bioconductor package.

### Data set

The CRC proteomes, 95 samples and 7210 proteins were downloaded from [42]; the data were already normalized and TCGA identifiers as well as clinical information were available for each sample [42]. CMS labels were matched to TCGA samples using CMS subtyping calls (file cms_labels_public_all.txt, (http://www.synapse.org/#!Synapse:syn2623706/wiki/) [40]. Out of 95 samples, 86 had CMS labels, 19 were from CMS1, 35 from CMS2, 15 from CMS3 and 17 from CMS4 tumors.

## Results

**Fig 1:**
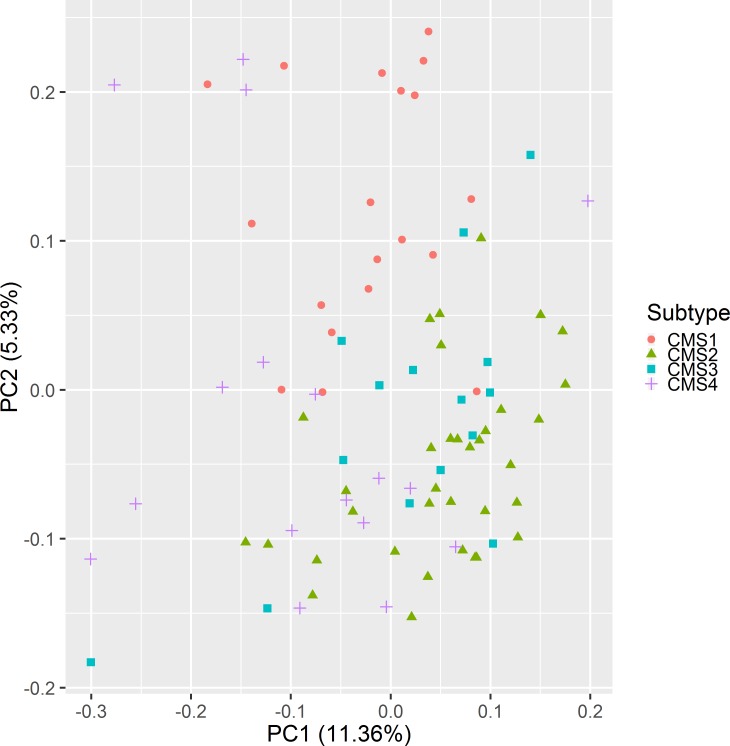
PCA plot for colon cancer subtypes. The number in parentheses indicates the percent of variance, explained by PC component. Only CMS1 is clearly separated from the rest.

When the samples for all four CMSs (N = 86) were analyzed by PCA based on their proteome features, the separation of subtypes was rather poor, with only CMS1 visibly separated from the rest (Fig 1). We therefore set to find out if there are any protein-level KEGG pathways (167 in MSigDB C2 collection) that were differentially expressed between CMSs.

### Proteome-level differences between CMSs of CRC

#### Competitive GSA tests

Neither ROMER nor GSEA detected significantly differentially expressed pathways at the level that was used for KS and ROAST (*p*_*adj*_<0.01). For GSEA, nothing was significant at *p*_*adj*_<0.01 for all comparisons (minimum *p*_*adj*_ value was .027). For ROMER, nothing was significant at *p*_*adj*_<0.01 as well. Minimum *p*_*adj*_ value was 0.027 for CMS1-CMS2 comparison and minimum *p*_*adj*_ value was 0.020 for CMS1-CMS234 comparison.

#### Self-contained GSA tests

**Fig 2:**
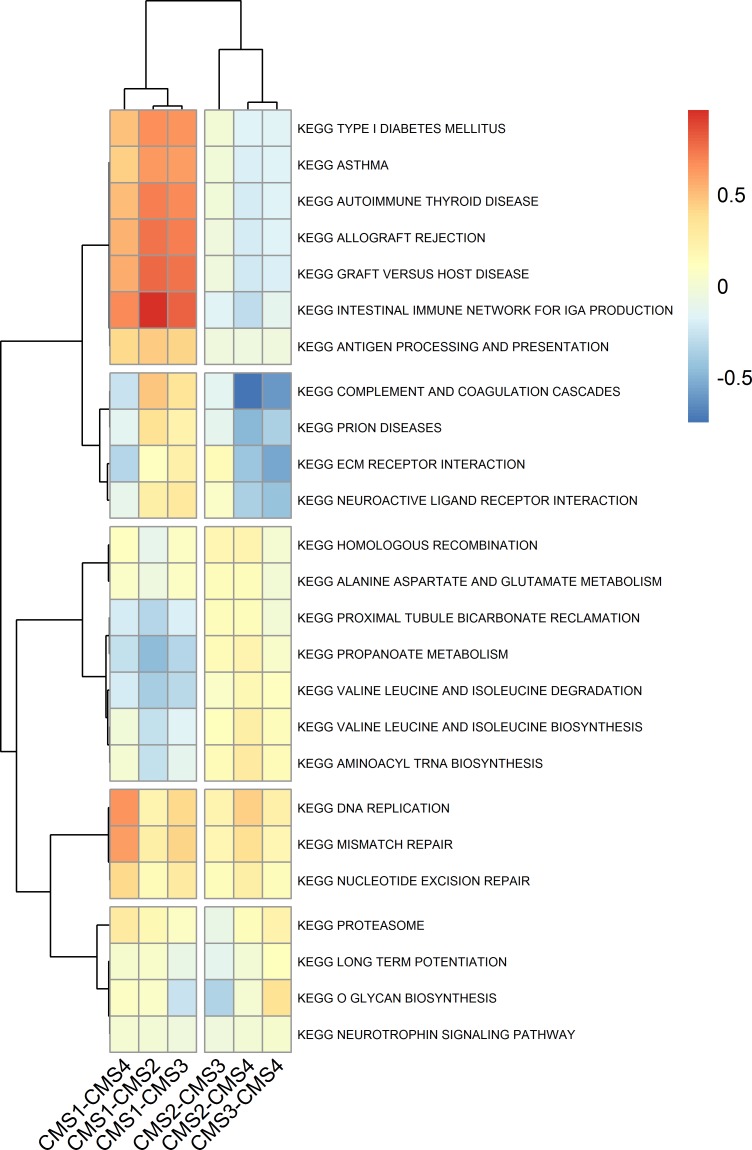
Heatmap of CRC subtypes, based on the protein-level pathways, differentially expressed between subtype pairs.

Fig 2 presents clustering of CRC subtypes, based on the protein-level pathways, differentially expressed between subtype pairs. The pathways were identified using ROAST test, after correction for multiple testing at the significance level *p*_*adj*_<0.01 and several additional requirements (see Methods section). Both pathways and pairs of subtypes were clustered using correlation distance and average abundance levels for each subtype pair. The subtype pairs were clearly separated into two groups–one included CMS1 compared with CMS2, CMS3 and CMS4 and another one included pairs of other three subtypes (Fig 2). It is not unexpected, given that according to transcriptomic classification CMS1 subtype was the most different from the rest of the tumor samples in terms of their molecular characteristics, namely being MSI+, hypermutated and with low frequency of CIN [40,41].

**Fig 3:**
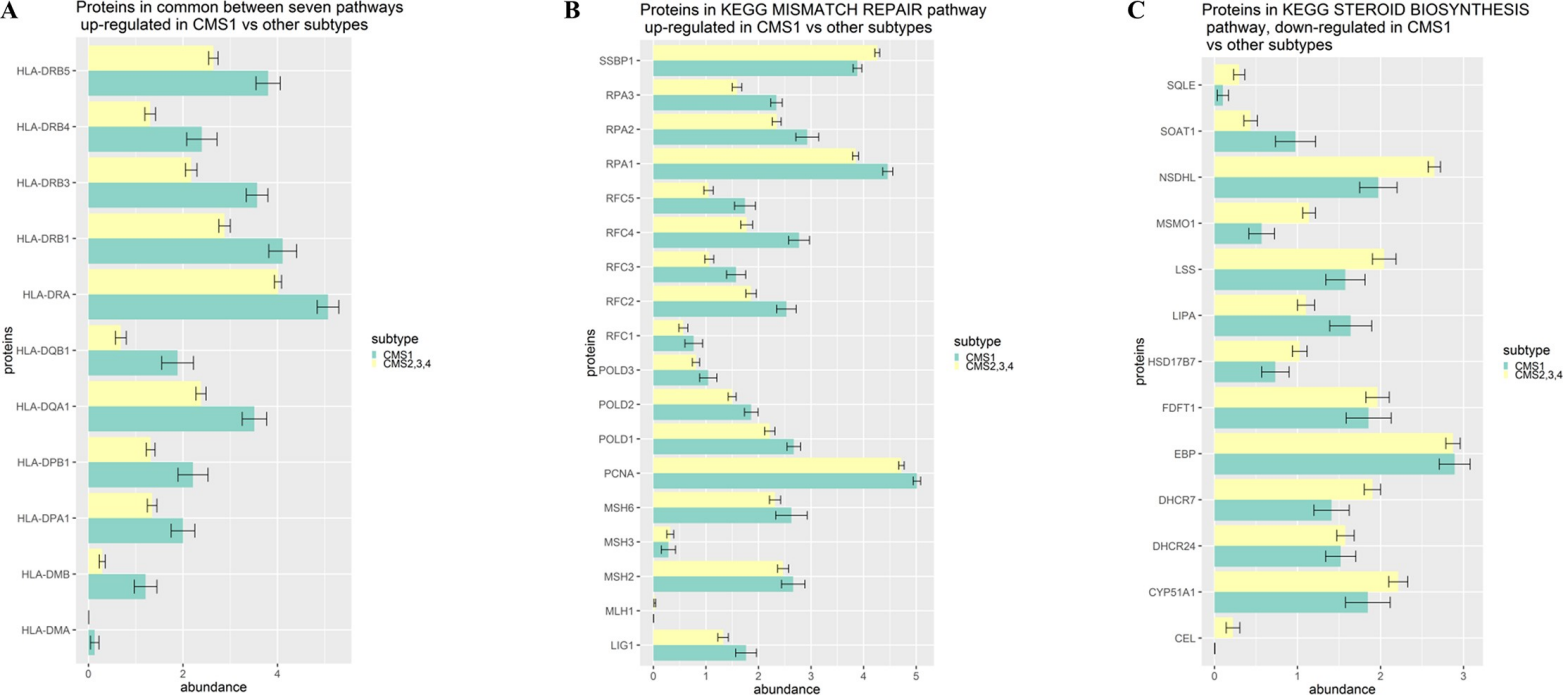
Differences between CMS1 and other CRC subtypes. A. Protein abundance of common proteins in the intersection of seven KEGG pathway for CMS1. All proteins are part of major histocompatibility complex (MHC) class II. B. Protein abundance in mismatch repair pathway for CMS1 and other subtypes. C. Protein abundance in steroid biosynthesis pathway for CMS1 and other subtypes.

**Pathways, differentially expressed between CMS1 and other CRC subtypes.** Table 1 presents pathways, differentially expressed between CMS1 and all other subtypes. The first seven pathways (Table 1) up-regulated in CMS1 as compared to the rest of samples were seemingly unrelated to CMS1 phenotype. However a closer look did show that these 7 pathways had 11 proteins in common, all of them were parts of major histocompatibility complex (MHC) class II (Fig 3A).

**Table 1:** ROAST results. Pathways differentially expressed between CMS1 and other subtypes.

KEGG Pathway	NGenes Originally	NGenes	PropUP	PropDown	Direction	FDR.Mixed
KEGG intestinal immune network for IgA production	48	14	0.93	0.00	Up UP	0.001
KEGG allograft rejection	38	20	0.85	0.00	UP	0.001
KEGG graft versus host disease	42	19	0.84	0.00	Up	0.001
KEGG asthma	30	16	0.81	0.18	UP	0.001
KEGG autoimmune thyroid disease	53	21	0.81	0.00	UP	0.001
KEGG type I diabetes mellitus	44	22	0.72	0.04	UP	0.001
KEGG antigen processing and presentation	89	46	0.65	0.08	UP	0.001
KEGG proteasome	48	40	0.67	0.05	UP	0.001
KEGG mismatch repairt	23	18	0.66	0.05	UP	0.001
KEGG DNA replication	36	31	0.64	0.03	UP	0.001
KEGG steroid biosynthesis	17	13	0.15	0.61	DOWN	0.001

MHC class II binds antigenic peptides and ‘presents’ them to antigen-specific CD4^+^ T-cells, thus stimulating their activation and differentiation into T helper cell subsets [52], while the MHC class I presents self-proteins for recognition by CD8^+^ cytotoxic T-cells. Under normal physiological conditions, peptides from autologous proteins do not trigger response because of the tolerance of CD8^+^ cytotoxic T-cells. However, neoantigenic peptides, which are generated by proteolysis of peptides with tumor-specific sequence alternations, may be recognized by CD8^+^ cytotoxic T cells, leading to elimination of cells with neo-epitopes. It has been known for a while that the mutational loads in DNA mismatch-repair system (MMR)-deficient MSI tumors are much higher than that in microsatellite stable (MSS) colorectal cancers. Presumably, MMR defect results in large amounts of neoantigens leading to higher immunogenicity of MSI tumors and their infiltration with CD8^+^ T cells and CD4^+^ T helper (T_H_1) cells [41,53]. In this hostile microenvironment, MSI-CRCs survive by overexpressing several immune checkpoint related proteins, including PD-1, PD-L1, CTLA-4, LAG-3 and IDO, at levels much higher than that in MSS-CRCs [54]. As a consequence, MSI-CRC patients are typically responding to immune checkpoint inhibitors [54].

The finding of eleven protein components of MHC class II at the intersection of seven pathways, involved in adaptive immune response (Table 1) suggest that CMS1 MSI-CRC tumor cells could potentially express MHC class II. In particular, both HLA-DRA and HLA-DRB1,3,4,5 were upregulated in CMS1 as compared to other CRC subtypes (Fig 3A), indicating the possibility that MSI-CRC tumors can present antigens with an aid of HLA-DR, a premier antigen-presenting MHC-II molecule. Notable, a majority of tumor cell types do not express MHC class II, and, because of that, escape direct elimination by cytotoxic CD4^+^ T cells [55]. Recently, a novel melanoma subtype with expression of MHC class II in general, and HLA-DR molecules in particular, was shown to be more responsive to the therapy targeting PD-1 [56]. Similarly to melanoma, the HLA-DR expression on MSI-CRC tumors could serve as a biomarker for selecting anti-PD-1 responsive patients and point toward a direction not yet explored in the context of MSI-CRCs.

Three other pathways, up-regulated in CMS1 as compared to CMS2, CMS3 and CMS4 subtypes, were ‘KEGG mismatch repair’, ‘KEGG proteasome’ and ‘KEGG DNA replication’. These findings are unexpected ones, as CMS1 is characterized by MSI with defects in MMR genes (MLH1, MSH2, MSH6 or PMS2) [57], and one would expect down-regulation of ‘KEGG mismatch repair’ pathway in CMS1. However, ROAST test found MMR pathway to be up-regulated in CMS1 as compared to other subtypes (Table 1). Indeed, for most of the MMR proteins, abundance was higher in CMS1 as compared to other subtypes (Fig 3B). Observed up-regulation of MMR proteins, even those with functional defects, along with similar trends in DNA replication and proteasome pathways may be explained as an attempt of the cell to compensate for intrinsically high mutation load, resulting in increased need for DNA repair, replication of the cells still capable of cell division, and proteolysis of neoantigenes. Interestingly, levels of MLH1 protein were low in all CRC subtypes (Fig 3B), not only in CMS1 where its inherited or acquired aberrations are the most common [57].

The only pathway down-regulated in CMS1 as compared to other subtypes was ‘KEGG steroid biosynthesis’, with expression of almost all proteins being lower in CMS1 as compared to other subtypes (Fig 3C).

According to analysis performing in a ROAST framework, the descriptions of CMS1 subtype at the transcriptome level [40,41] and at proteome levels (present work) matched very closely. In addition, self-contained GSA analysis of CMS1 proteome provided additional insights into molecular background of CMS1, in particular, allowing us to uncover its potential for expressing HLA-DR.

**Pathways, differentially expressed between CMS2 and other subtypes.** CMS2 is considered to be canonical CRC subtype. Table 2 presents pathways which were differentially expressed between CMS2 and other subtypes of CRC as identified by ROAST. Not surprisingly, the pathways that were up-regulated in CMS1, including the common set of MHC-II related proteins, were predominantly down-regulated in CMS2, in agreement with previously described non-immunogenic transcriptome signature of CMS2 [40,41].

**Table 2:** ROAST results. Pathways differentially expressed between CMS2 and other subtypes.

KEGG pathway	NGenes Originally	NGenes	PropUP	PropDown	Direction	FDR.Mixed
KEGG intestinal immune network for IGA production	48	14	0.00	0.78	DOWN	0.001
KEGG allograft rejection	38	20	0.00	0.75	DOWN	0.002
KEGG graft versus host disease	42	19	0.00	0.78	DOWN	0.002
asthma	30	16	0.12	0.62	DOWN	0.002
KEGG autoimmune thyroid disease	53	21	0.00	0.71	DOWN	0.002
KEGG type I diabetes mellitus	44	22	0.04	0.68	DOWN	0.001
KEGG complement and coagulation cascade	69	57	0.01	0.77	DOWN	0.001
KEGG valine leucine and isoleucine biosynthesis	11	10	0.70	0.10	UP	0.001
KEGG valine leucine and isoleucine degradation	44	43	0.62	0.00	UP	0.001
KEGG propanoate metabolismKEGG proximal tubule bicarbonate reclamation	3323	2916	0.680.62	0.10 0.00	UPUP	0.0010.001
KEGG aminoacyl tRNA biosynthesis	41	38	0.62	0.00	UP	0.001

Interestingly, both the synthesis and the degradation pathways for branched-chain amino acid (BCAA: leucine, valine and isoleucine) were up-regulated in CMS2 as compared to other subtypes of CRC (Table 2). While it is well known that tumor growth depends on amino acids, especially BCAAs, and their preferential uptake by tumors reported previously [58,59], up-regulation of anabolic and catabolic BCAA pathways in either CMS2 or any other CRC subtypes had not been yet noted. Up-regulation of ‘KEGG aminoacyl tRNA biosynthesis’ (Table 2), which was also observed in CMS2 tumors, was previously found to be a general property of cancer cells [60]. Here, the up-regulation of this pathway could be a byproduct of increased utilization of BCAA which fuels a protein biosynthesis. Similarly, an increase in the levels of proteins comprising ‘KEGG proximal tubule bicarbonate reclamation’ pathway may be tied to BCAA utilization and protein biosynthesis through a necessity to counterbalance metabolic acidosis associated with tumor growth and increased turnover of proteins.

Another interesting pathway up-regulated in CMS2 was ‘KEGG propionate metabolism’. As one of microbiome produced short chain fatty acids (SCFAs), propionate considered to have beneficial effect on colon physiology. It has been shown that microbial production of propionate may be stimulated by lactate which is secreted by CRC cells in course of glycolysis [61]. Finding that CMS2 tumors differentially express the proteins of propionate pathways may possibly provide a functional link between CRCs and associated dysbiosis [62].

The analysis of the CMS2 CRC proteome subtype with ROAST did not point toward up-regulation of WNT and MYC downstream targets, or increased levels of cyclins observed at the transcriptome level [40,41]. Instead, this analysis highlighted novel actionable pathways and a novel set of candidate protein biomarker molecules capable of identifying patients with CMS2 tumors.

**Pathways, differentially expressed between CMS3 and other subtypes.** At the given level of significance, no pathways were differentially expressed between CMS3 and other subtypes of CRC (see ‘**Additional CMS-specific insights extracted using GSNCA’** section below).

**Fig 4:**
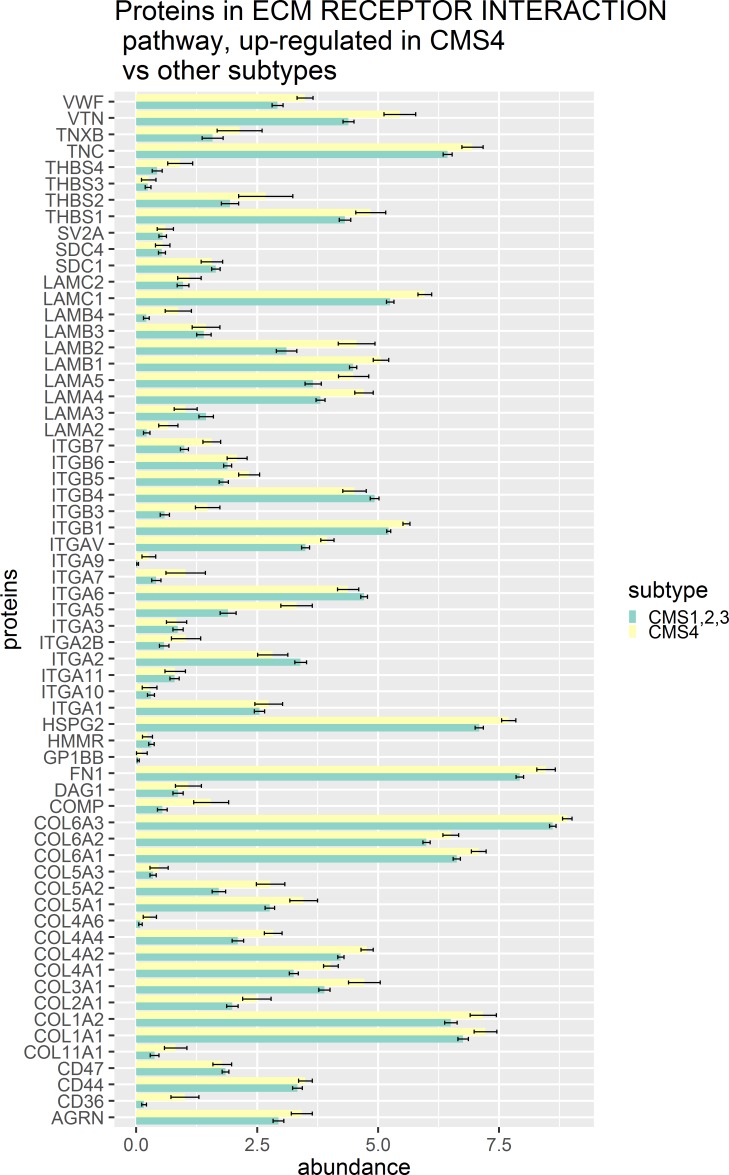
Protein abundance in ECM receptor interaction pathway for CMS4 and other subtypes.

**Pathways, differentially expressed between CMS4 and other subtypes.** In ROAST analysis, a total of five pathways, were differentially expressed between CMS4 and other subtypes of CRC (Table 3). First, virtually all members of ‘KEGG ECM receptor interaction’ pathway were up-regulated in CMS4 as compared to other subtypes (Fig 4). This pathway includes collagens, integrins, thrombospondin, fibronectin and other proteins dynamically involved in the formation of extracellular matrix and the epithelial-mesenchymal transition (EMT) [63–65]. Overall, the role of extracellular matrix in EMT is well acknowledged [66,67], and observed up-regulation of the EMT related pathway is in agreement with CMS4 transcriptomic signature [40,41]. Similarly, in CMS4 the complement-mediated inflammatory system was up-regulated both at the transcriptome [40] and at the proteome levels (Table 3). It should be noted that CMS4-specific up-regulation of “KEGG prion diseases” was secondary to the complement up-regulation; out of 25 proteins present in prion-related pathway, all 25 were also the members of the compliment pathway.

**Table 3:** ROAST results. Pathways differentially expressed between CMS4 and other subtypes.

KEGG Pathway	NGenes Originally	NGenes	PropUP	PropDown	Direction	FDR.Mixed
KEGG ECM receptor interaction	84	63	0.65	0.00	UP	0.001
KEGG prion diseases KEGG complement and coagulation cascades	3569	2557	0.600.87	0.080.00	UPUP	0.0010.001
KEGG mismatch repair	23	18	0.00	0.66	DOWN	0.001
KEGG DNA replication	36	31	0.00	0.62	DOWN	0.001

### Additional CMS-specific insights extracted using GSNCA

In GSNCA analysis, five pathways were differentially co-expressed between CMS1 and CMS3, eleven pathways were differentially co-expressed between CMS2 and CMS1,3,4 and 2 pathways were differentially co-expressed and sorting out CMS3 from CMS1,2,4 (S1 Fig). GSNCA did not find any differentially co-expressed pathways between CMS4 and other subtypes. Significant pathways were those with Benjamini-Hochberg adjusted p-value <0.1. Below we will consider pathways differentially co-expressed between CMS3 and CMS1,2,4 in a greater detail.

**Fig 5:**
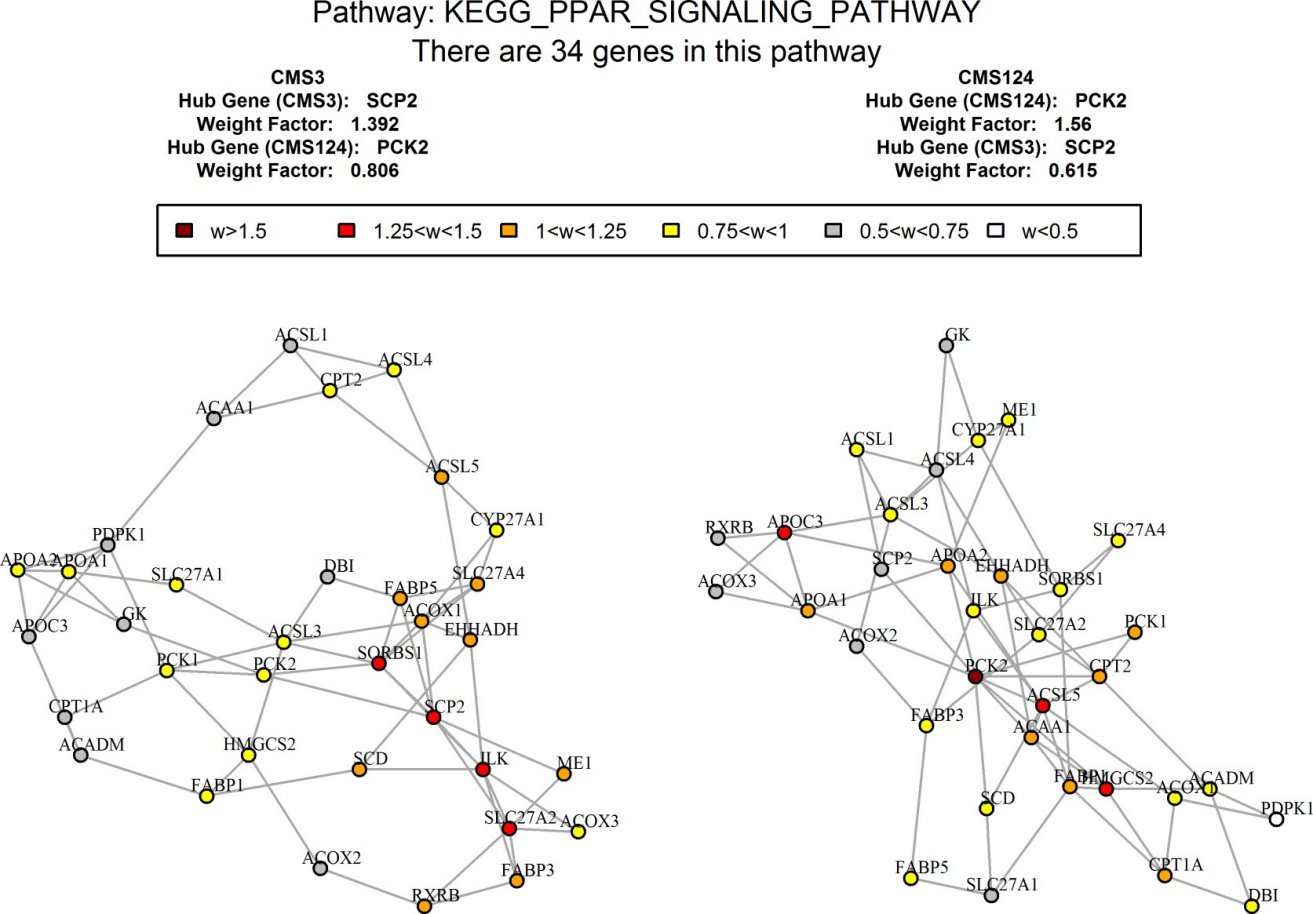
Different co-expression network configurations of PPAR signaling pathway in CMS3 vs other subtypes. Major changes in network’s hub proteins (in red).

**Pathways, differentially co-expressed between CMS3 and other subtypes.** Previous transcriptome analyses showed that the major feature of CMS3 CRC subtype is metabolic reprogramming, including activation of glutaminolysis and lipidogenesis [40,41]. In proteome analysis, the top differentially co-expressed pathway to separate CMS3 from the rest of CRCs was ‘KEGG PPAR signaling pathway’ (Fig 5). This pathway includes three types of peroxisome proliferator-activated receptors, namely PPARalpha, beta/delta, and gamma, which orchestrate lipid metabolism, lipid oxidation and cell proliferation, or adipocyte differentiation to enhance blood glucose uptake, respectively. Notably, only a part of PPAR signaling pathway was upregulated in CRCs, this part specifically excluded the PPAR receptors. The difference between CMS3 and the other subtypes was in PPAR co-expression networks configuration. In particular, the cholesterol transport proteins (sterol carrier protein 2, SCP2) and fatty acid binding protein-1 (FATP1 or SLC27A1), ILK (integrin-linked kinase) and SORBS1 (the sorbin and SH3 domain containing 1) proteins were highly interconnected in CMS3 subtype, but not in the other CRCs (Fig 5). Integrin-linked kinase (ILK) performs crucial roles in the control of human intestinal cell and crypt-villus axis homeostasis, as well as intestinal cell proliferation, spreading, and migration [68]. Since a majority of cholesterol is stored in plasma membrane [69], CMS3-specific coordinated changes in levels of ILK and cholesterol transporters may point at cholesterol trafficking, and subsequently altered cholesterol distribution, as important contributors to CMS3 phenotype. Cholesterol-driven modification of integrin signaling and resultant changes in the extracellular matrix cell may explain relatively poor prognosis associated with CMS3 subtype of CRC.

**Fig 6:**
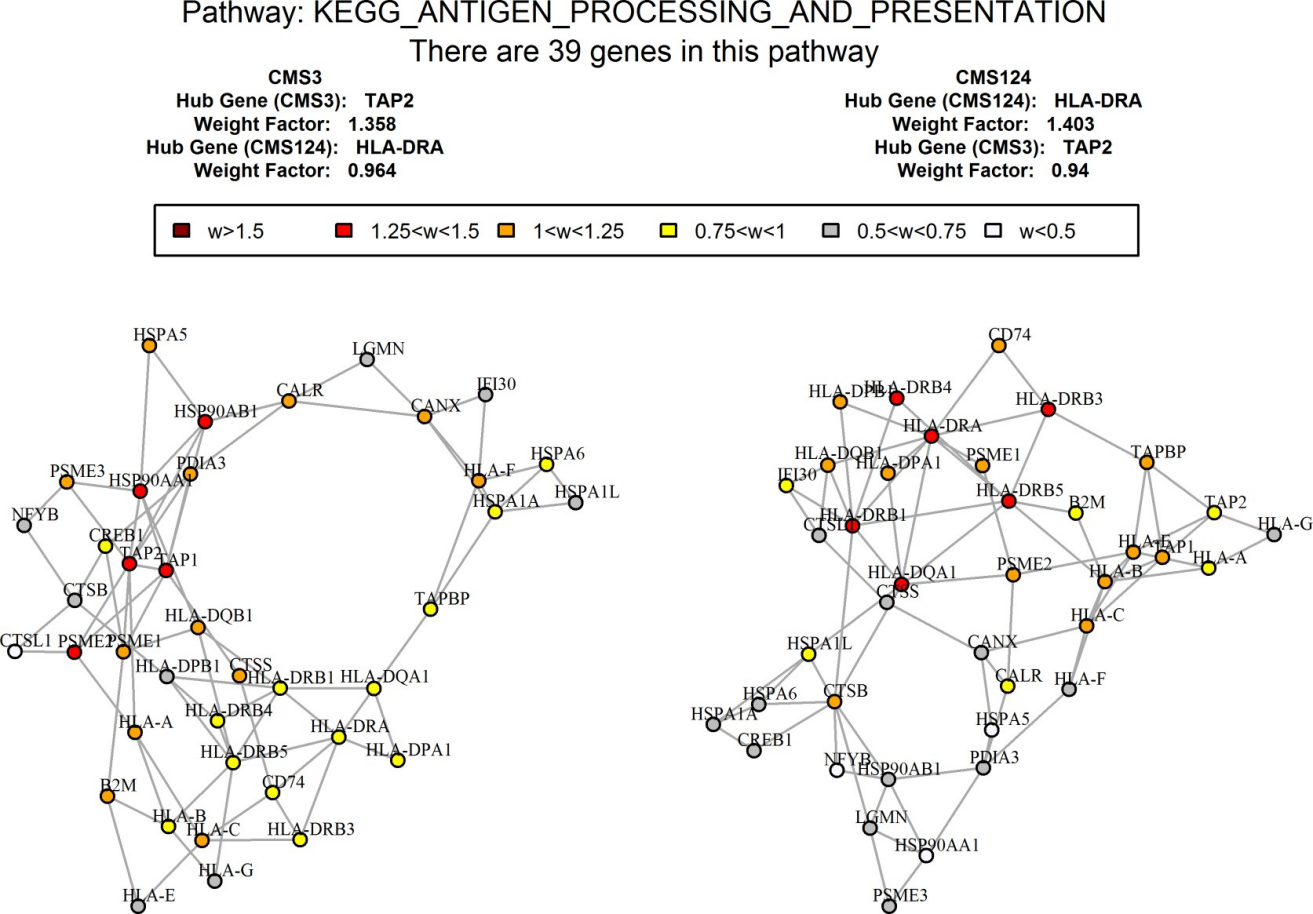
Different co-expression network configurations of antigen signaling pathway in CMS3 vs other subtypes. Major changes in network’s hub proteins (in red).

Another differentially co-expressed pathway between CMS3 and the rest of CRCs was ‘KEGG antigen processing and presentation’ (Fig 6). For CMS3, the hub protein of this pathway was TAP2, a transporter associated with antigen processing, while for the other subtypes the pathway was centered on HLA-DRA, which serves as a part of primary antigen-presenting MHC-II complex. Differential regulation of antigen processing and presentation pathway likely reflects characteristic features of CMS3 subtype, namely its suppressed immune and inflammatory signatures [40,41].

Overall, a set of proteome-derived pathways de-regulated in CMS3 subtype was in agreement with already known characteristics of CMS3 CRCs, with an important addition of a novel CMS3-specific molecular target, a trafficking of cholesterol.

## Discussion

There are many excellent bioinformatics methods developed for proteomics data, including the techniques for normalization and preprocessing (e.g. spectral counts modeling with edgeR [70], MetaMass [71], reviewed in [72]), detecting and quantifying protein complexes (CCprofiler [73]), protein-protein interaction networks analysis and visualization (Cytoscape [74]) as well as dedicated software platforms with a set of statistical tools for high-dimensional proteomics data analysis (e.g. Perseus [75]). There are even competitive GSA tests specifically developed for proteomics data [19,20]. Intriguingly, there are no self-contained GSA approaches specifically developed for proteomics data analysis, as well as to the best of our knowledge no attempts were made to apply existing transcriptomics self-contained GSA tests to proteomics data. Given that self-contained GSA approaches have more power than competitive ones, it is reasonable to assume that proteomics data analysis may be aided by adoption of self-contained GSA tests previously developed for transcriptomics.

To investigate this possibility, we applied several self-contained GSA approaches, namely KS, RKS [46], ROAST [47] and GSNCA [46] to proteome profiles matched with consensus molecular subtype (CMS) labels, previously derived from transcriptomic data of colorectal cancers [40]. In total, we analyzed 86 proteome samples classified into four CMSs.

For several reasons, exact similarity between transcriptome and proteome-based portraits of CMSs may not be expected. Proteomics data are different from transcriptomics data as a consequence of a combination of molecular properties of proteins and technological challenges. First, after mRNA is synthesized, post-transcriptional, translational and protein degradation regulation take place and control steady-state protein abundances [43]. In both bacteria and eukaryotes, correlation between protein and mRNA abundances is at approximately a squared Pearson correlation coefficient of ~0.40, i.e. only 40% of the variation in protein abundance may be explained by abundance of respective mRNAs [43]. As it would not be enough, despite the power of contemporary MS-based technologies some parts of the proteome remain hidden as a consequence of proteins physicochemical characteristics and technological biases [76]. Despite all those challenges, quite surprisingly, many characteristic features of CMSs found at the mRNA level were reproduced at the protein level with self-contained GSA tests. It should be noted, that competitive GSA tests did not have enough power to find differentially expressed pathways at the given level of significance.

Using ROAST framework, we found that proteome of CMS1 subtype was most different from that of other subtypes of CRC. Similar to its transcriptome signature, proteome-level molecular portrait of CMS1 features heavy involvement of the components of immune system as well as the pathways related to mismatch repair, DNA replication and functioning of proteasome. A new observation visible at the proteome, but not at the transcriptome level, was the abundance of MHC-II related proteins, indicating possible involvement of MHC-II antigens in the known immunogenicity of CMS1. This observation might have clinical implications, as it could be used as additional indicator for the likelihood of tumor response to anti-PD-1 therapy, similar to that observed in MHC-II expressing melanomas [56].

CMS4 subtype was originally defined as ‘inflammatory’ and ‘mesenchymal-like’, with detected upregulation of complement pathway and genes involved in epithelial-to-mesenchymal transition (EMT) [40,41]. These observations were replicated at the protein level. In particular, we found an upregulation of both ECM receptor interactors and components of complement pathway. Because this upregulation was observed at the protein level, some of the included proteins might likely be converted to actionable targets for the treatment of mesenchymal CRCs of CMS4. Indeed, previous studies of various colon carcinoma models showed that the suppression of EMT may be achieved by integrin (included in ECM receptor interaction pathway) interaction disruptors [77]. Other ECM receptor interaction pathway proteins (Fig 4) could potentially be good candidates for targeted therapy.

For CMS2, proteome-level descriptors were different from mRNA signatures. Proteome portrait of CMS2 was reminiscent of ‘garden-variety’ pan-tumor signature augmented by notable upregulation of the metabolism of branched chain amino acids, propionate and amino acyl t-RNA synthesis. Given the aforementioned incongruence of transcriptome and proteome data some pathways are expected to be identified at proteome level only. These additional pathways may serve as a source of CMS2-specific metabolic biomarkers, capable of tracing the tumor burden.

For CMS3, also known as ‘metabolic’ subtype, an analysis with ROAST framework failed to detect any differentiating protein-level pathways. However, when the same dataset was analyzed with GSNCA, two differentially co-expressed CMS3 specific pathways were detected. One of them, PPAR signaling, was critically rearranged to emphasize on a connection between cholesterol trafficking and the regulation of kinase ILK. This observation may pave the way to CMS3-specific therapies aimed at the metabolism of cholesterol, for example, with lipid-lowering drugs.

Overall, the use of self-contained GSA approaches allow reconciliation of the insights derived from the molecular portraits of tumor subtypes independently built on transcriptomics and proteomics levels for colon cancer data. Moreover, protein level self-contained GSA approaches are capable of highlighting additional molecular pathways and actionable targets, which are visible at the protein level only. In the future, it would be beneficial to complement proteomics data analysis with self-contained GSA tests, in addition to competitive tests specifically developed for proteomics data.

## Supporting information

S1 Fig All pathways, differentially co-expressed between CMSs. (PDF)
