# Supplementary material for: Proteome-transcriptome alignment of molecular portraits achieved by self-contained gene set analysis: Consensus colon cancer subtypes case study
Source: PLoS One. 2019 Aug 22;14(8):e0221444. doi: 10.1371/journal.pone.0221444 (PMC6705791; doi:10.1371/journal.pone.0221444)

Pathway: KEGG_ANTIGEN_PROCESSING_AND_PRESENTATION
There are 39 genes in this pathway

CMS1
Hub Gene (CMS1): HLA−DRA
Weight Factor: 1.365
Hub Gene (CMS3): TAP2
Weight Factor: 0.899

CMS3
Hub Gene (CMS3): TAP2
Weight Factor: 1.358
Hub Gene (CMS1): HLA−DRA
Weight Factor: 0.964

w>1.5 | 1.25<w<1.5 | 1<w<1.25 | 0.75<w<1 | 0.5<w<0.75 | w<0.5

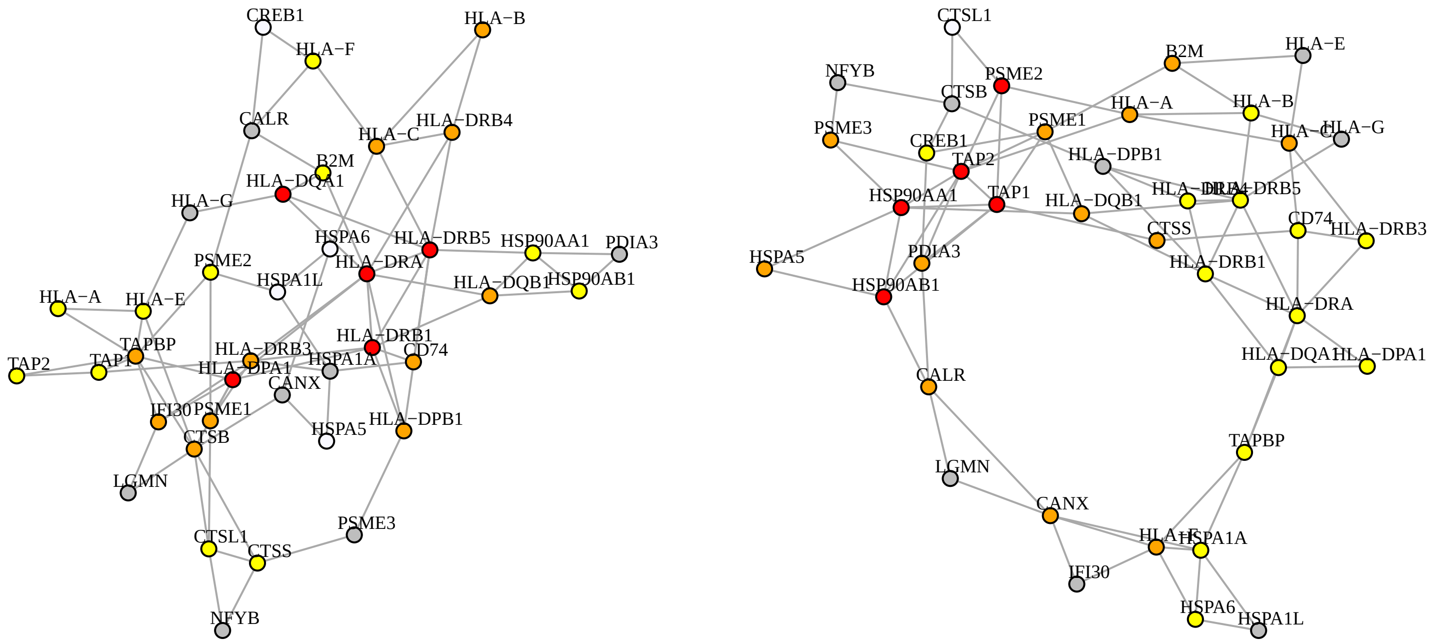

Pathway: KEGG_CYTOSOLIC_DNA_SENSING_PATHWAY
There are 18 genes in this pathway

CMS1
Hub Gene (CMS1): DDX58
Weight Factor: 1.345
Hub Gene (CMS3): IL18
Weight Factor: 0.818

CMS3
Hub Gene (CMS3): IL18
Weight Factor: 1.264
Hub Gene (CMS1): DDX58
Weight Factor: 0.777

w>1.5 | 1.25<w<1.5 | 1<w<1.25 | 0.75<w<1 | 0.5<w<0.75 | w<0.5

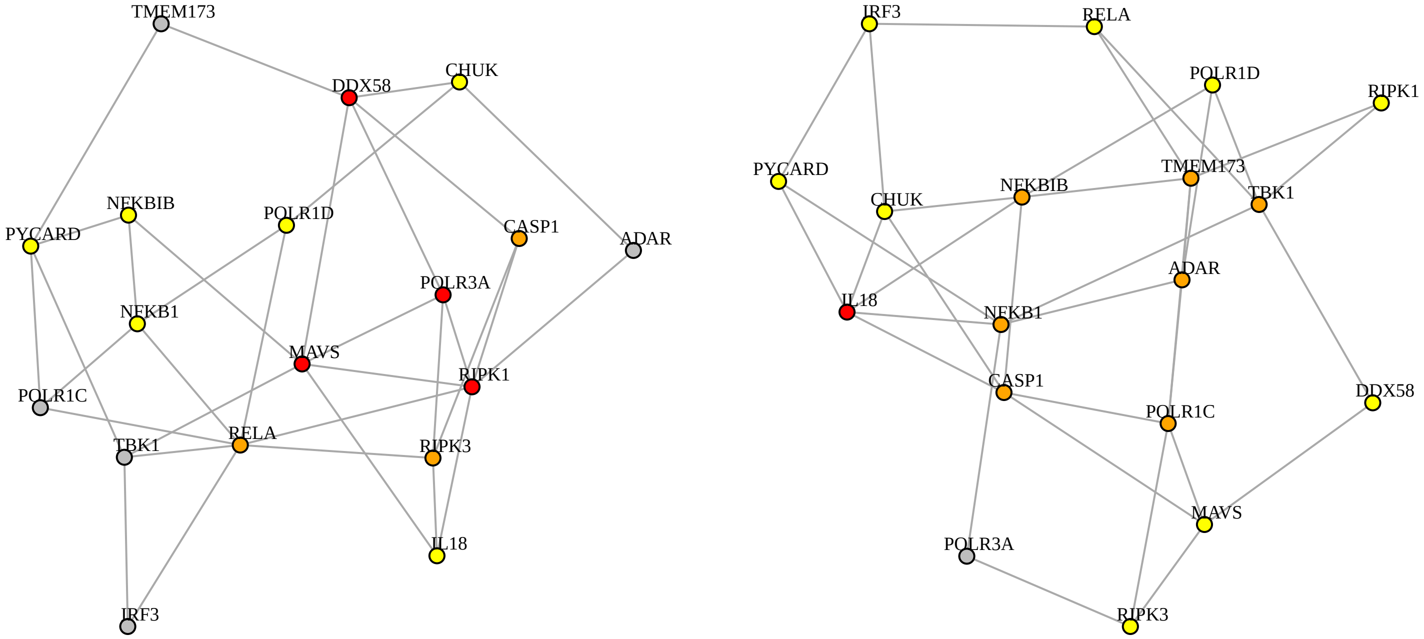

Pathway: KEGG_PATHWAYS_IN_CANCER
There are 122 genes in this pathway

CMS1
Hub Gene (CMS1): MAPK9
Weight Factor: 1.344
Hub Gene (CMS3): MSH2
Weight Factor: 0.953

CMS3
Hub Gene (CMS3): MSH2
Weight Factor: 1.458
Hub Gene (CMS1): MAPK9
Weight Factor: 0.883

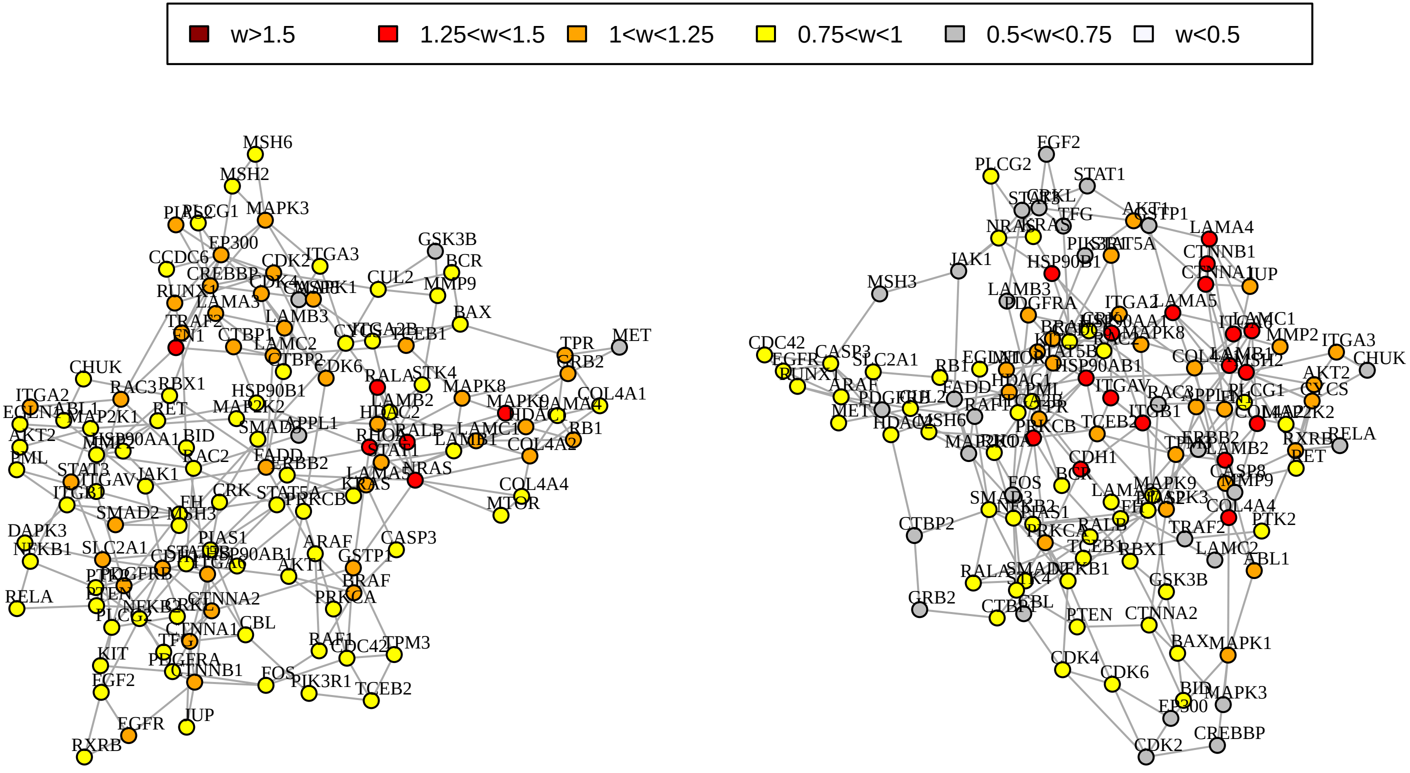

Pathway: KEGG_SMALL_CELL_LUNG_CANCER
There are 36 genes in this pathway

CMS1
Hub Gene (CMS1): FN1
Weight Factor: 1.385
Hub Gene (CMS3): LAMC1
Weight Factor: 1.235

CMS3
Hub Gene (CMS3): LAMC1
Weight Factor: 1.344
Hub Gene (CMS1): FN1
Weight Factor: 1.189

w>1.5	1.25<w<1.5	1<w<1.25	0.75<w<1	0.5<w<0.75	w<0.5

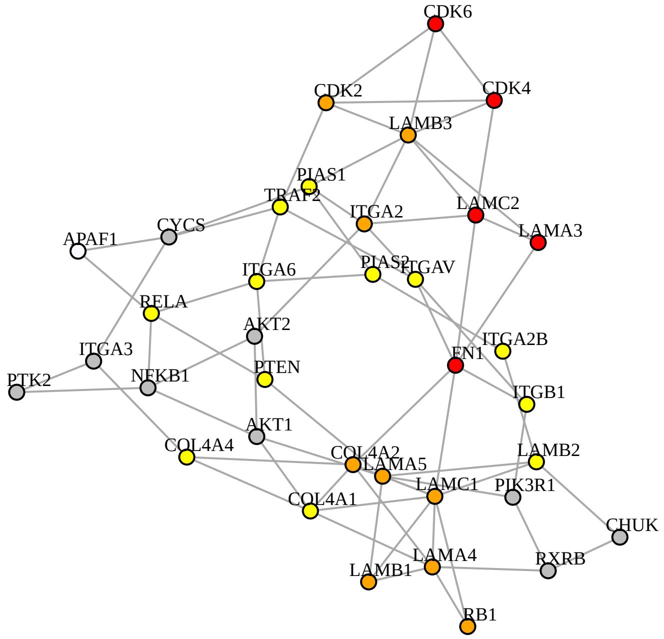

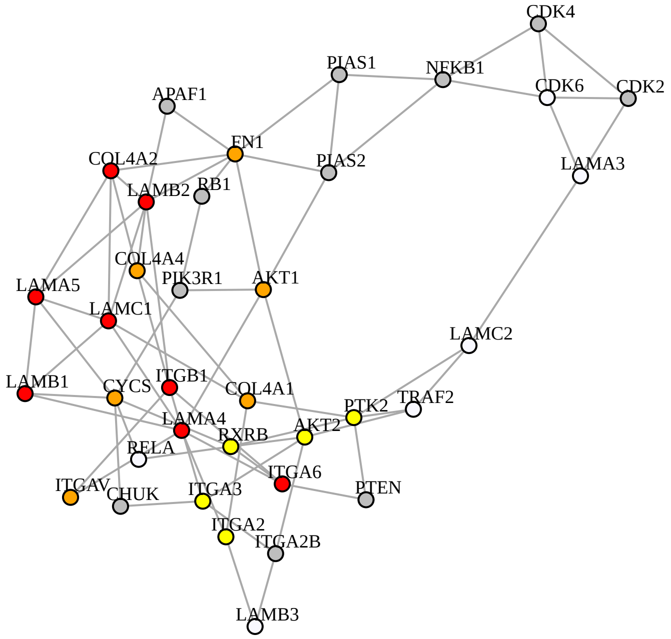

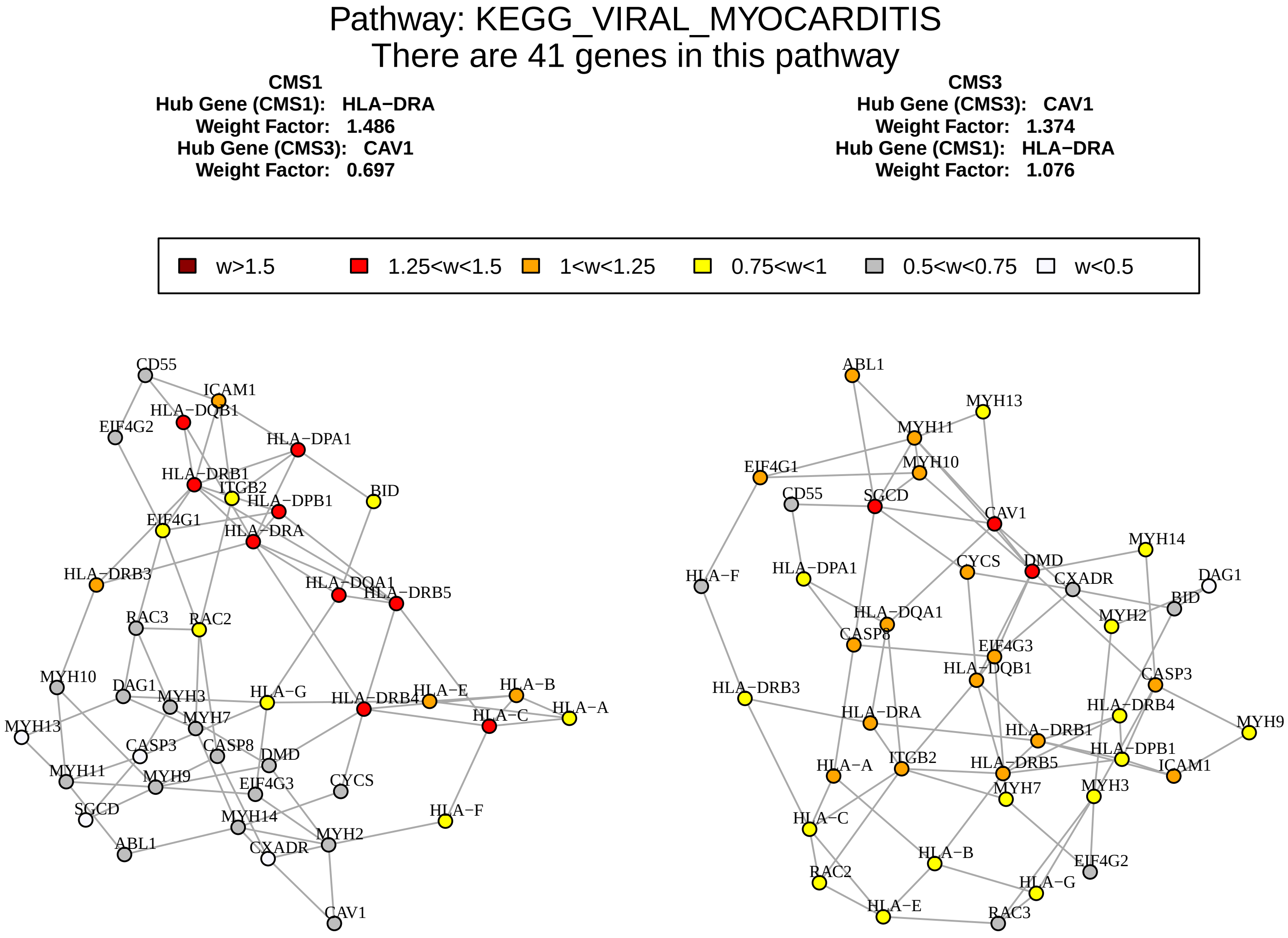
Pathway: KEGG_VIRAL_MYOCARDITIS
There are 41 genes in this pathway
CMS1
Hub Gene (CMS1): HLA−DRA
Weight Factor: 1.486
Hub Gene (CMS3): CAV1
Weight Factor: 0.697
CMS3
Hub Gene (CMS3): CAV1
Weight Factor: 1.374
Hub Gene (CMS1): HLA−DRA
Weight Factor: 1.076
w>1.5
1.25<w<1.5
1<w<1.25
0.75<w<1
0.5<w<0.75
w<0.5
CD55
ICAM1
HLA−DQB1
EIF4G2
HLA−DPA1
HLA−DRB1
ITGB2
HLA−DPB1
BID
EIF4G1
HLA−DRA
HLA−DRB3
HLA−DQA1
HLA−DRB5
RAC3
RAC2
MYH10
DAG1
MYH3
HLA−G
HLA−DRB4
HLA−E
HLA−B
HLA−A
HLA−C
MYH13
MYH7
CASP3
CASP8
DMD
MYH11
MYH9
EIF4G3
CYCS
SGCD
MYH14
HLA−F
ABL1
MYH2
CXADR
CAV1
ABL1
MYH13
MYH11
EIF4G1
MYH10
CD55
SGCD
CAV1
MYH14
CYCS
DMD
CXADR
DAG1
HLA−F
HLA−DPA1
BID
HLA−DQA1
MYH2
CASP8
EIF4G3
HLA−DQB1
CASP3
HLA−DRB3
HLA−DRB4
MYH9
HLA−DRA
HLA−DRB1
HLA−DPB1
ICAM1
HLA−A
ITGB2
HLA−DRB5
MYH7
MYH3
HLA−C
HLA−B
EIF4G2
RAC2
HLA−G
HLA−E
RAC3

Pathway: KEGG_PURINE_METABOLISM
There are 64 genes in this pathway

CMS2
Hub Gene (CMS2): POLD2
Weight Factor: 1.318
Hub Gene (CMS134): POLR2A
Weight Factor: 0.901

CMS134
Hub Gene (CMS134): POLR2A
Weight Factor: 1.483
Hub Gene (CMS2): POLD2
Weight Factor: 0.983

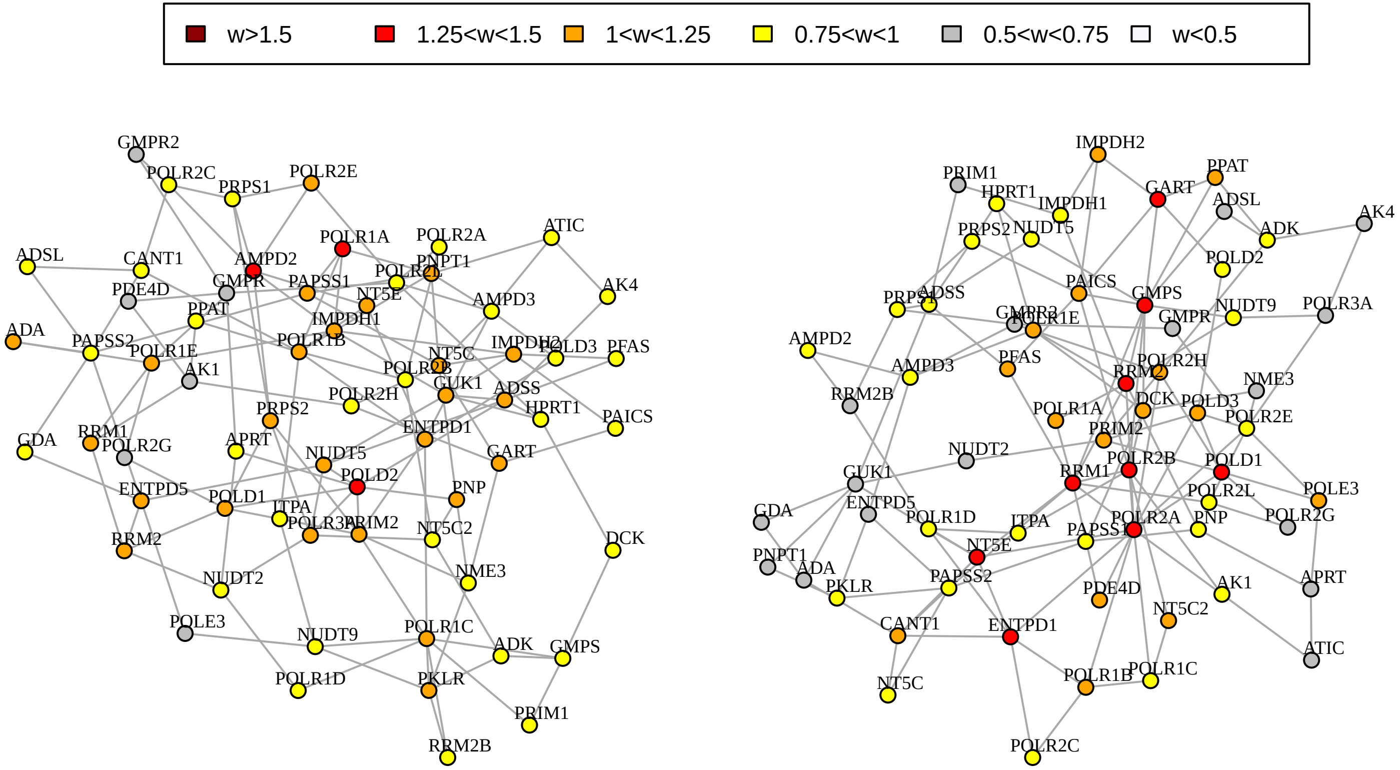

Pathway: KEGG_PROTEIN_EXPORT
There are 20 genes in this pathway

CMS2
Hub Gene (CMS2): SPCS1
Weight Factor: 1.425
Hub Gene (CMS134): SRP54
Weight Factor: 0.739

CMS134
Hub Gene (CMS134): SRP54
Weight Factor: 1.353
Hub Gene (CMS2): SPCS1
Weight Factor: 0.972

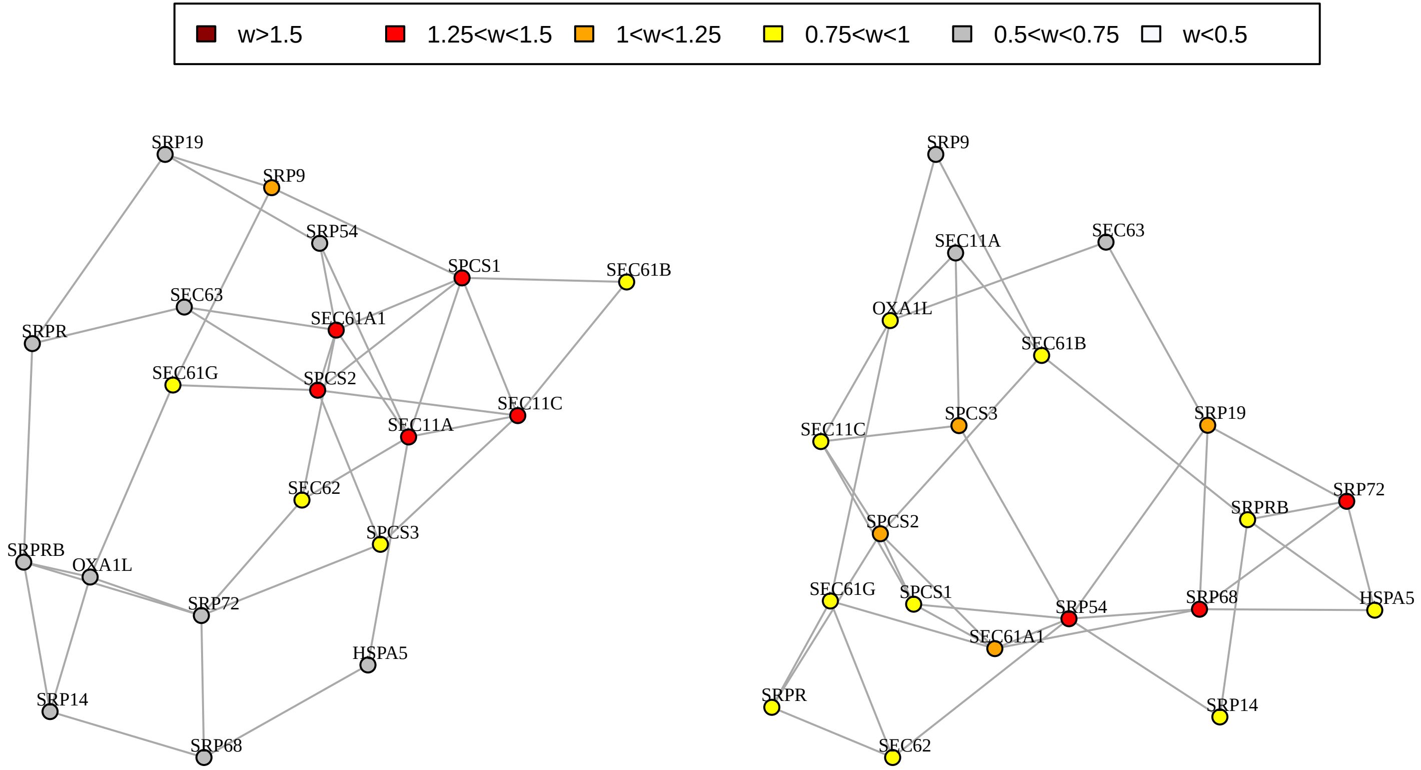

Pathway: KEGG_NUCLEOTIDE_EXCISION_REPAIR
There are 22 genes in this pathway

CMS2
Hub Gene (CMS2): PCNA
Weight Factor: 1.314
Hub Gene (CMS134): PCNA
Weight Factor: 1.314

CMS134
Hub Gene (CMS134): PCNA
Weight Factor: 1.455
Hub Gene (CMS2): PCNA
Weight Factor: 1.455

w>1.5 | 1.25<w<1.5 | 1<w<1.25 | 0.75<w<1 | 0.5<w<0.75 | w<0.5

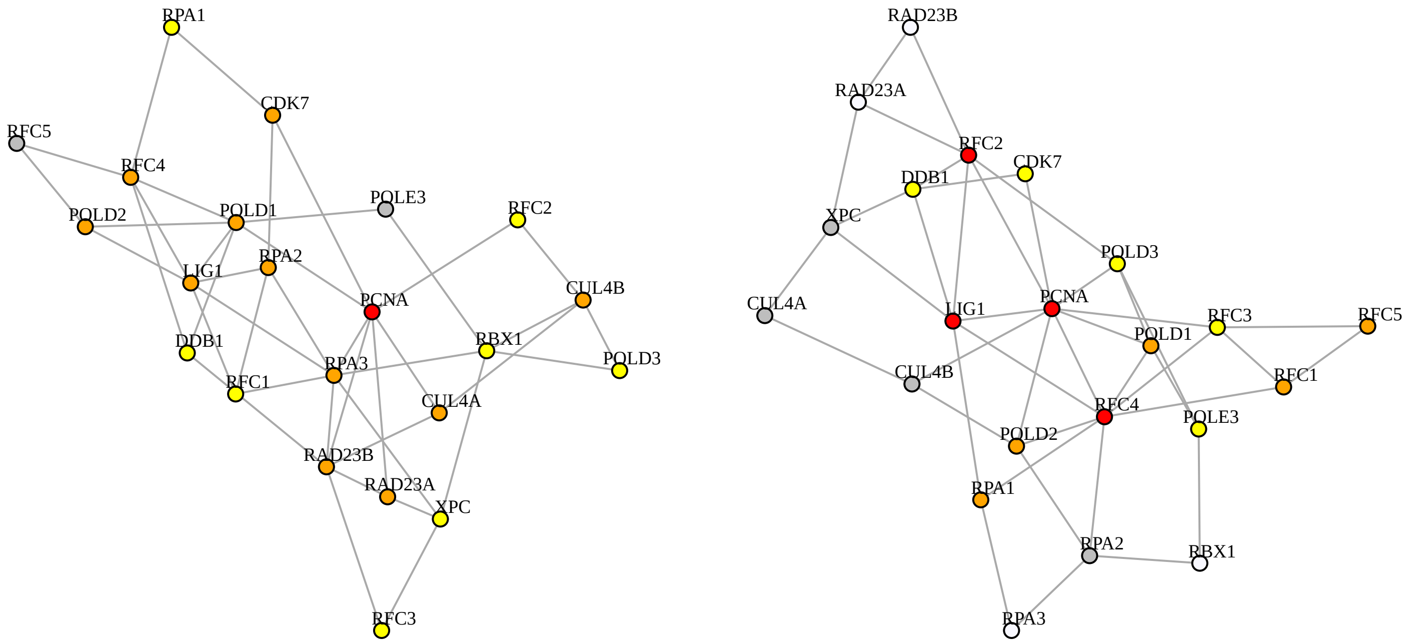

Pathway: KEGG_PEROXISOME
There are 41 genes in this pathway

CMS2
Hub Gene (CMS2): ACSL1
Weight Factor: 1.391
Hub Gene (CMS134): ACSL5
Weight Factor: 1.275

CMS134
Hub Gene (CMS134): ACSL5
Weight Factor: 1.408
Hub Gene (CMS2): ACSL1
Weight Factor: 0.767

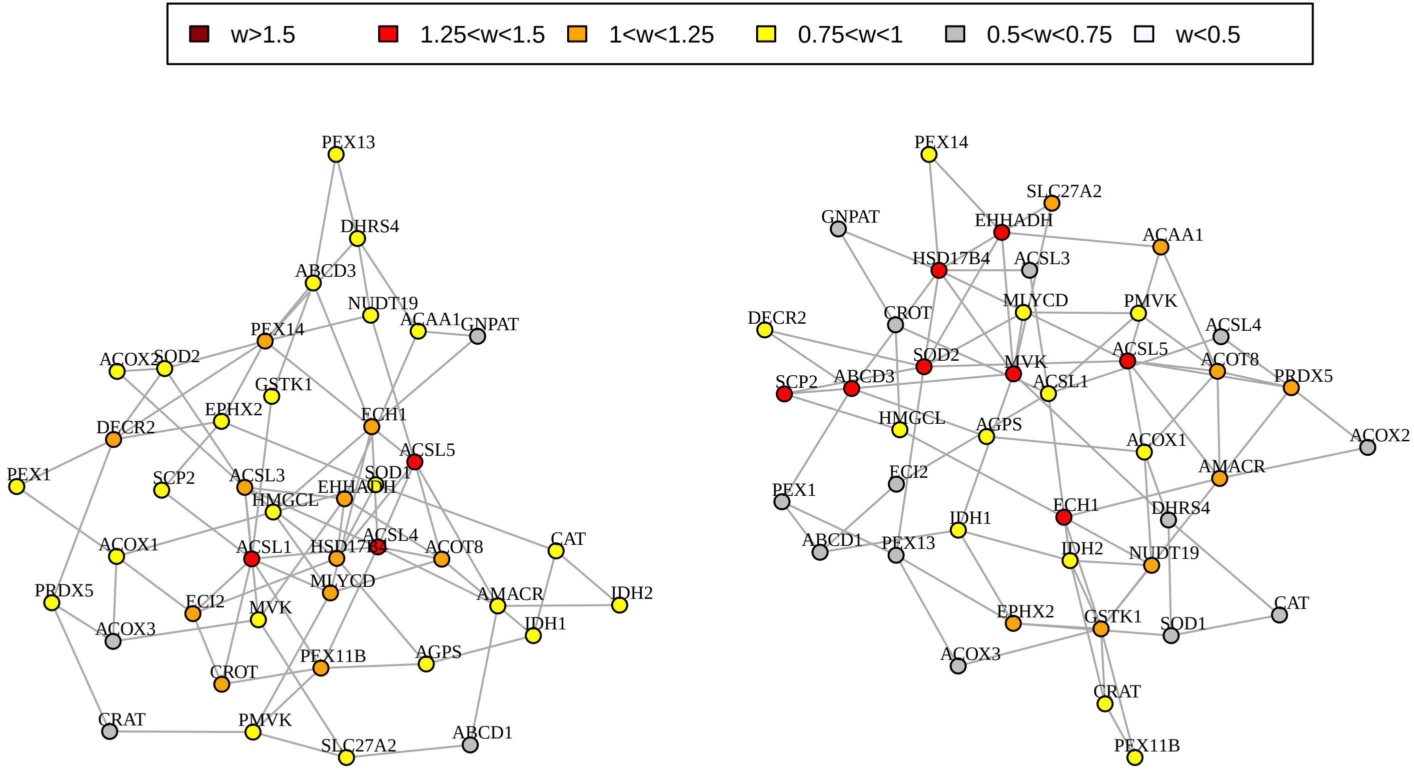

Pathway: KEGG_COMPLEMENT_AND_COAGULATION_CASCADES
There are 44 genes in this pathway

CMS2
Hub Gene (CMS2): KNG1
Weight Factor: 1.26
Hub Gene (CMS134): KNG1
Weight Factor: 1.26

CMS134
Hub Gene (CMS134): KNG1
Weight Factor: 1.191
Hub Gene (CMS2): KNG1
Weight Factor: 1.191

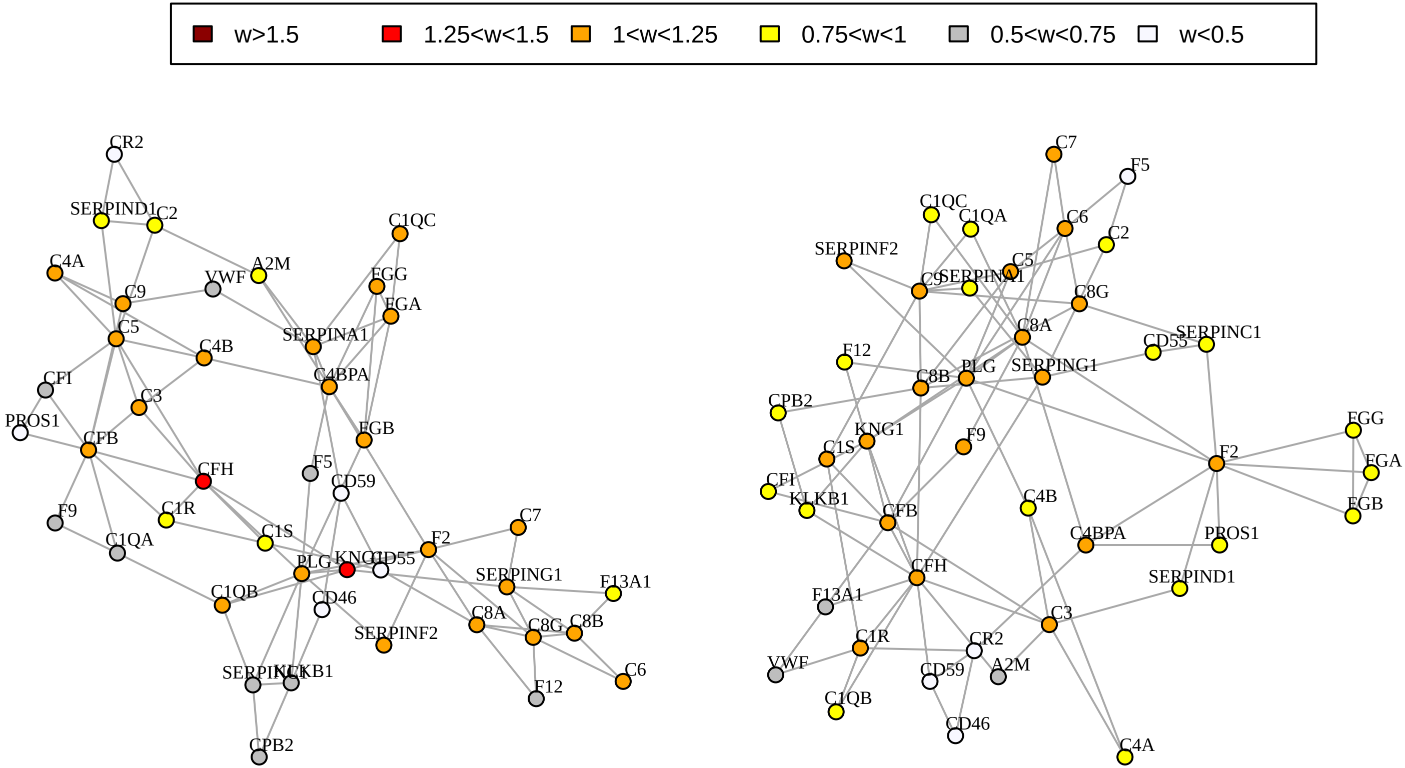

Pathway: KEGG_NATURAL_KILLER_CELL_MEDIATED_CYTOTOXICITY

There are 41 genes in this pathway

CMS2
Hub Gene (CMS2): MAPK3
Weight Factor: 1.41
Hub Gene (CMS134): HLA−B
Weight Factor: 1.277

CMS134
Hub Gene (CMS134): HLA−B
Weight Factor: 1.48
Hub Gene (CMS2): MAPK3
Weight Factor: 0.794

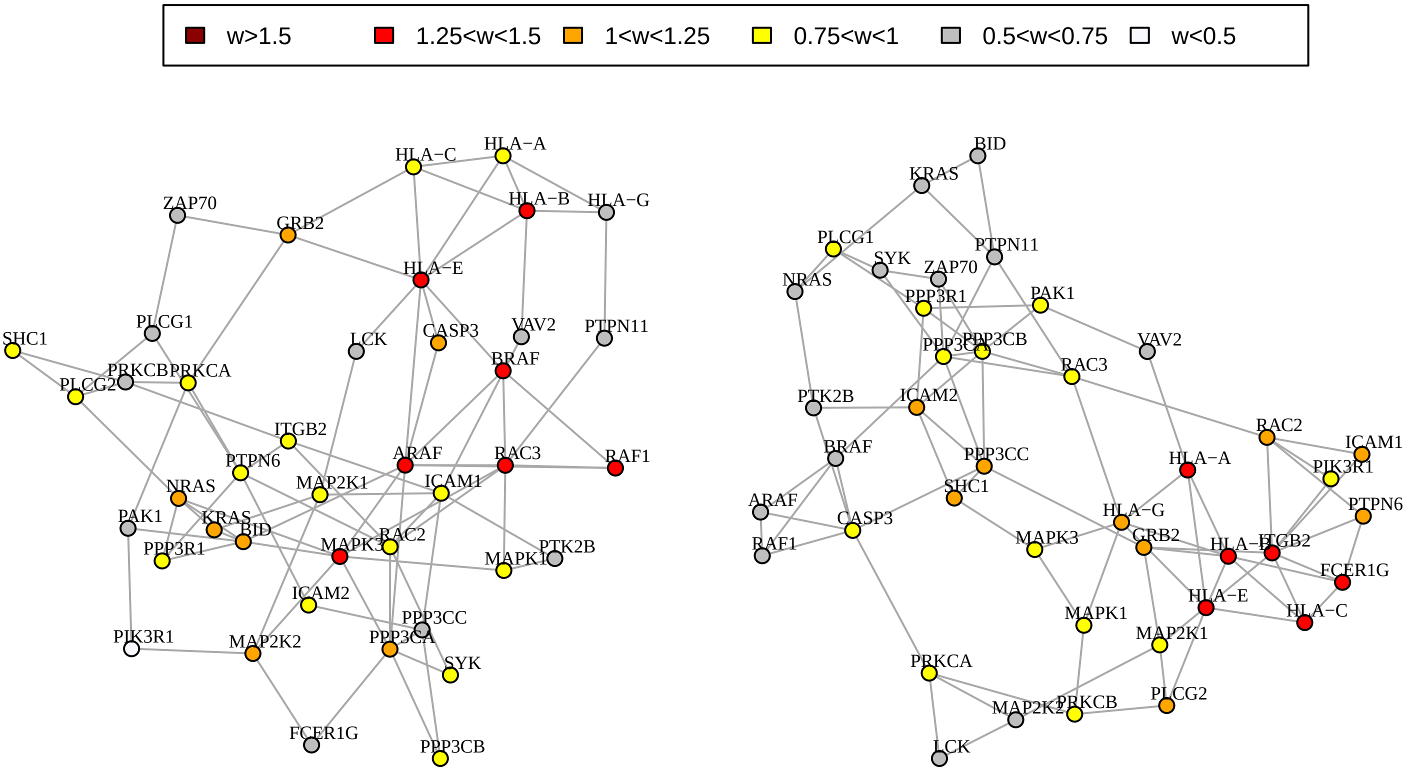

Pathway: KEGG_PRION_DISEASES
There are 23 genes in this pathway

CMS2
Hub Gene (CMS2): C8G
Weight Factor: 1.334
Hub Gene (CMS134): C8A
Weight Factor: 1.166

CMS134
Hub Gene (CMS134): C8A
Weight Factor: 1.328
Hub Gene (CMS2): C8G
Weight Factor: 1.199

w>1.5 | 1.25<w<1.5 | 1<w<1.25 | 0.75<w<1 | 0.5<w<0.75 | w<0.5

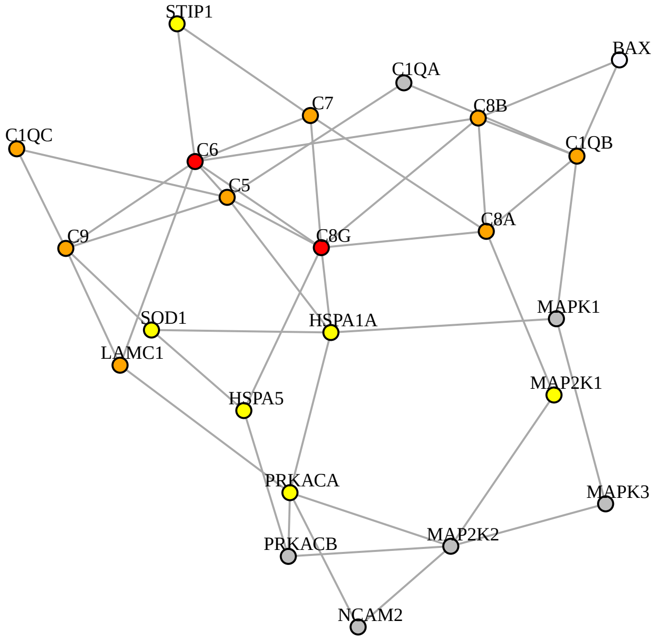

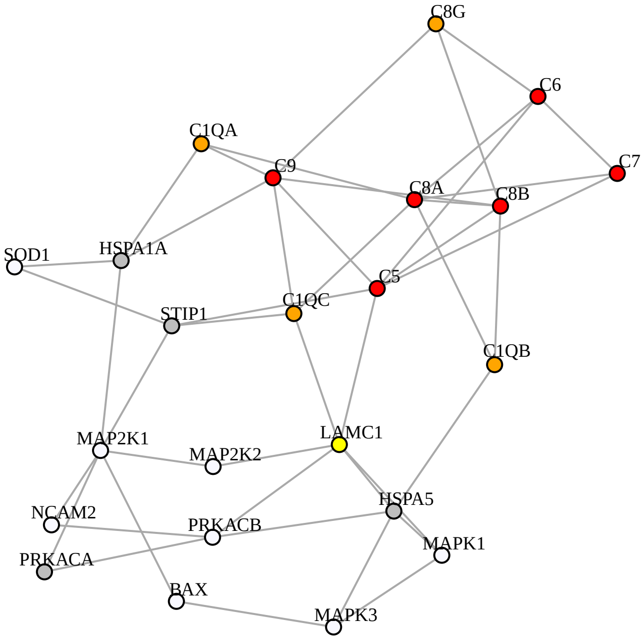

Pathway: KEGG_ENDOMETRIAL_CANCER
There are 23 genes in this pathway

CMS2
Hub Gene (CMS2): ARAF
Weight Factor: 1.381
Hub Gene (CMS134): CTNNA1
Weight Factor: 0.847

CMS134
Hub Gene (CMS134): CTNNA1
Weight Factor: 1.539
Hub Gene (CMS2): ARAF
Weight Factor: 0.704

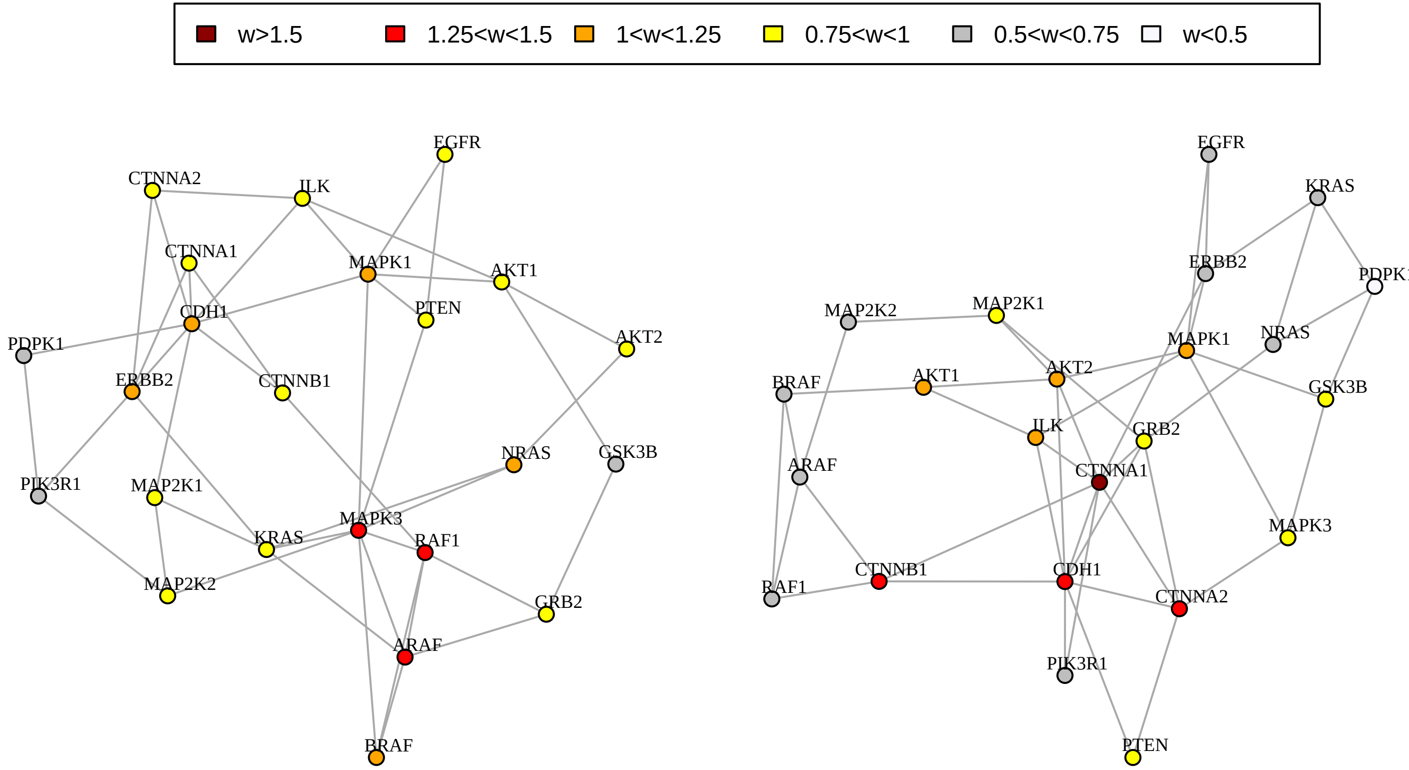

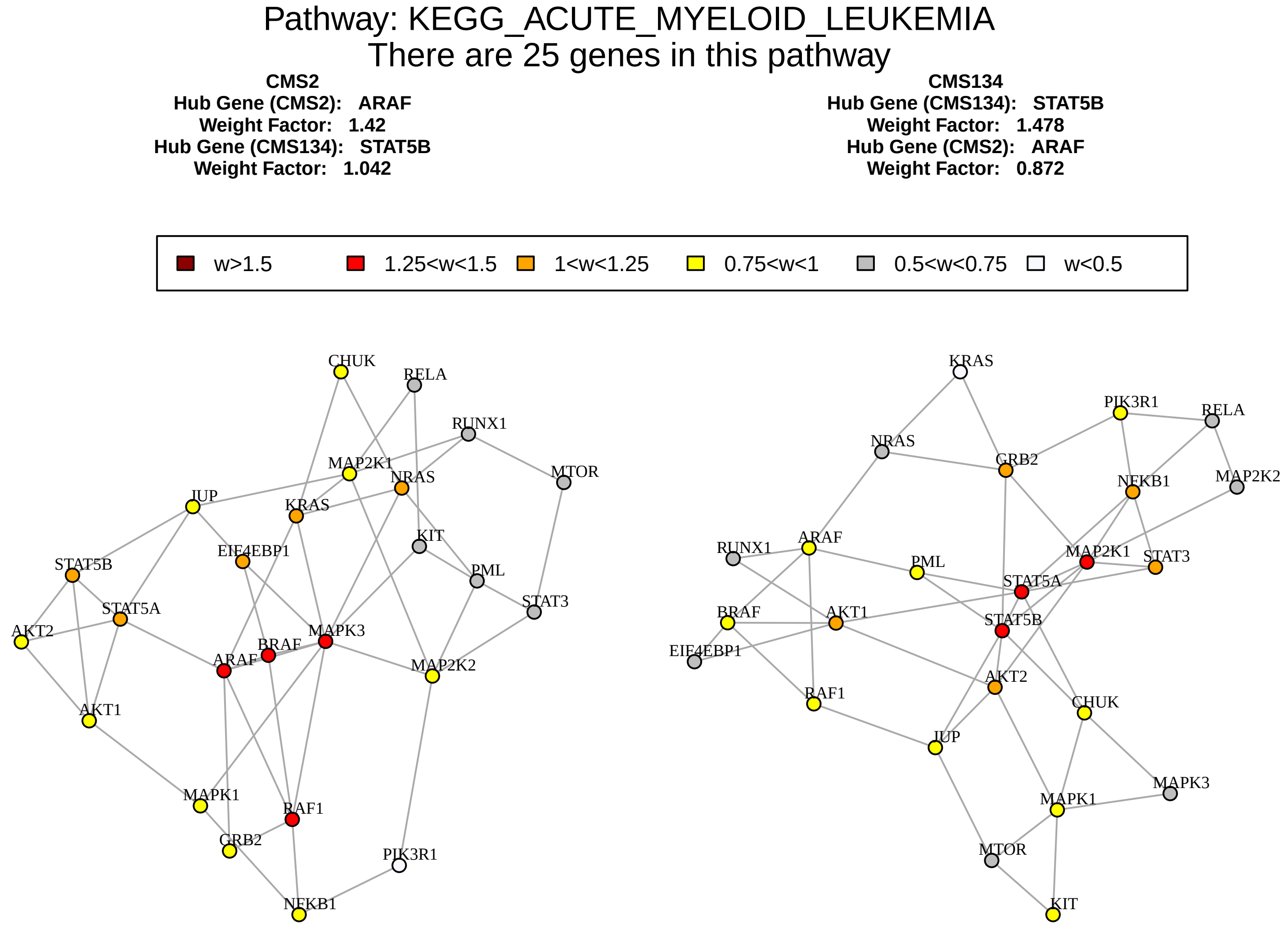

Pathway: KEGG_ACUTE_MYELOID_LEUKEMIA
There are 25 genes in this pathway
CMS2
Hub Gene (CMS2): ARAF
Weight Factor: 1.42
Hub Gene (CMS134): STAT5B
Weight Factor: 1.042
CMS134
Hub Gene (CMS134): STAT5B
Weight Factor: 1.478
Hub Gene (CMS2): ARAF
Weight Factor: 0.872
w>1.5
1.25<w<1.5
1<w<1.25
0.75<w<1
0.5<w<0.75
w<0.5
CHUK
RELA
RUNX1
MTOR
MAP2K1
NRAS
JUP
KRAS
KIT
EIF4EBP1
PML
STAT5B
STAT5A
STAT3
AKT2
MAPK3
BRAF
ARAF
MAP2K2
AKT1
MAPK1
RAF1
GRB2
PIK3R1
NFKB1
KRAS
PIK3R1
RELA
NRAS
GRB2
NFKB1
MAP2K2
ARAF
RUNX1
MAP2K1
STAT3
PML
STAT5A
BRAF
AKT1
STAT5B
EIF4EBP1
AKT2
RAF1
CHUK
JUP
MAPK3
MAPK1
MTOR
KIT

Pathway: KEGG_NON_SMALL_CELL_LUNG_CANCER
There are 25 genes in this pathway

CMS2
Hub Gene (CMS2): ARAF
Weight Factor: 1.497
Hub Gene (CMS134): PRKCB
Weight Factor: 0.537

CMS134
Hub Gene (CMS134): PRKCB
Weight Factor: 1.289
Hub Gene (CMS2): ARAF
Weight Factor: 0.919

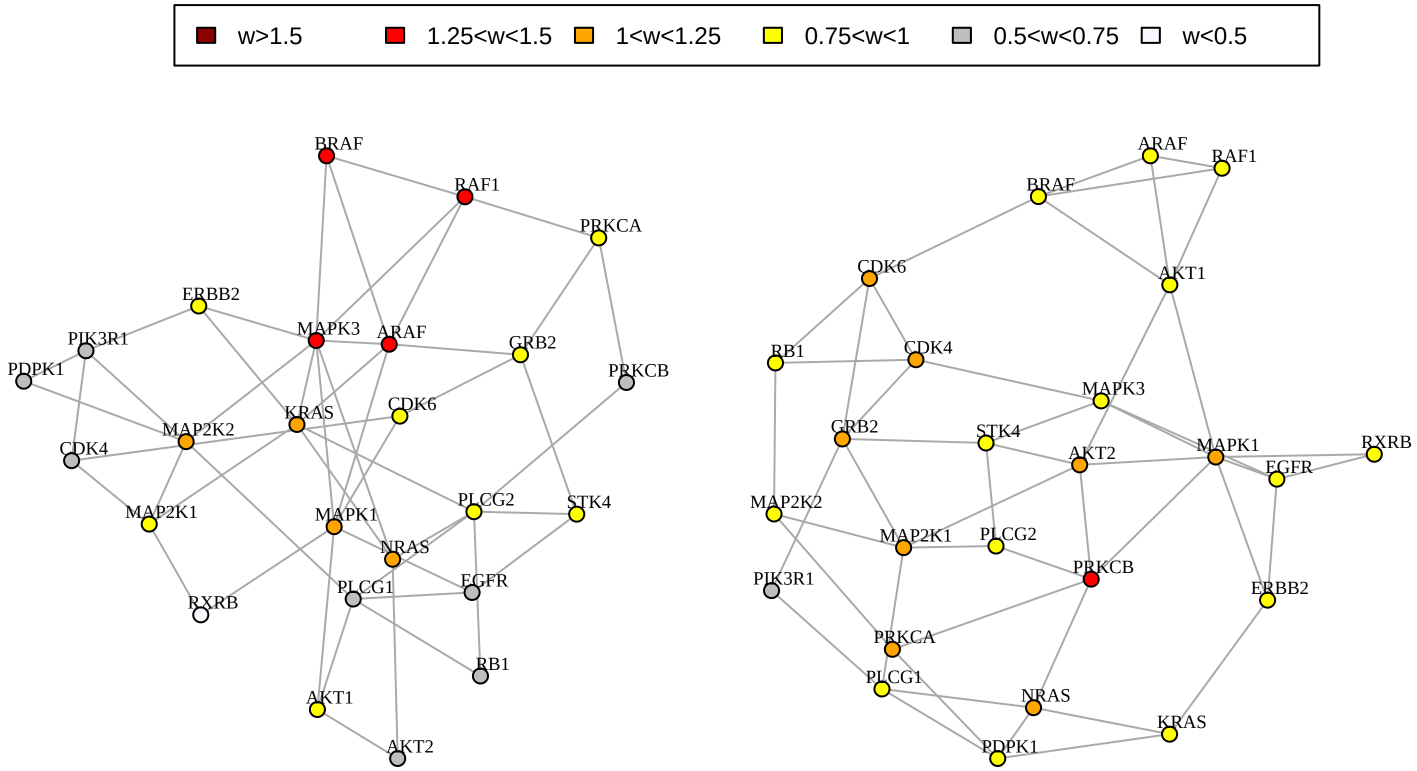

Pathway: KEGG_ASTHMA
There are 13 genes in this pathway

CMS2
Hub Gene (CMS2): HLA−DRA
Weight Factor: 1.258
Hub Gene (CMS134): HLA−DRA
Weight Factor: 1.258

CMS134
Hub Gene (CMS134): HLA−DRA
Weight Factor: 1.187
Hub Gene (CMS2): HLA−DRA
Weight Factor: 1.187

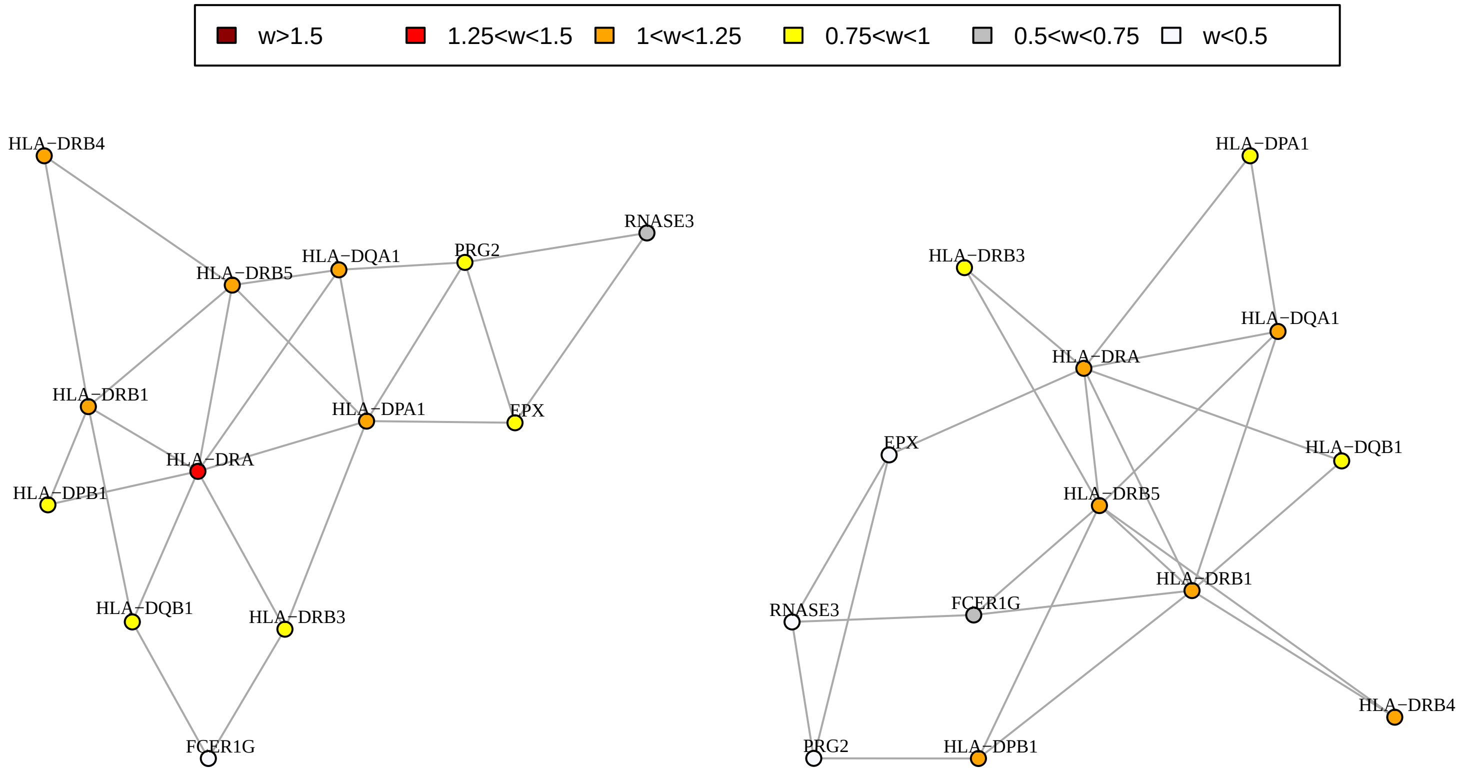

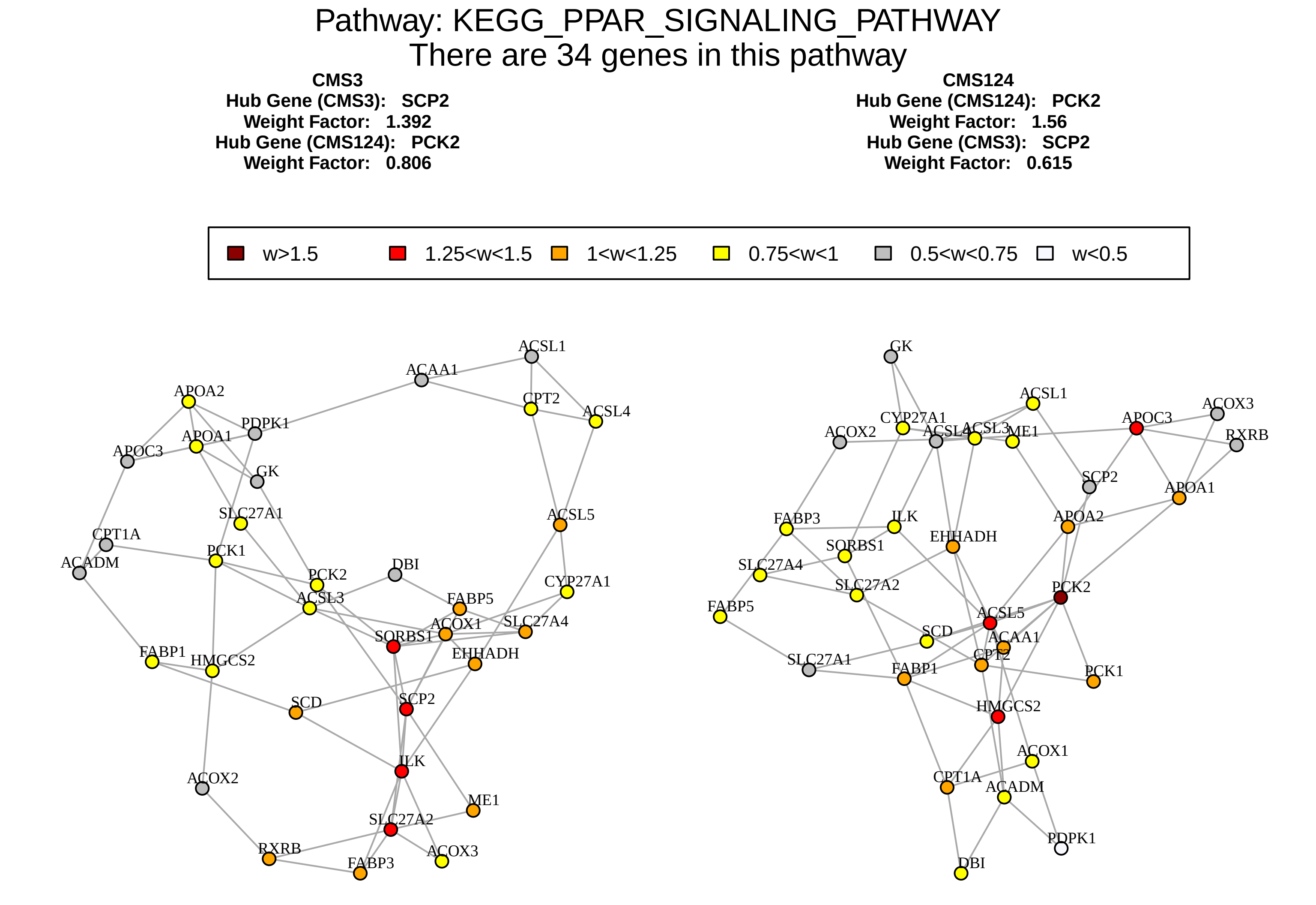
Pathway: KEGG_PPAR_SIGNALING_PATHWAY
There are 34 genes in this pathway
CMS3
Hub Gene (CMS3): SCP2
Weight Factor: 1.392
Hub Gene (CMS124): PCK2
Weight Factor: 0.806
CMS124
Hub Gene (CMS124): PCK2
Weight Factor: 1.56
Hub Gene (CMS3): SCP2
Weight Factor: 0.615
w>1.5
1.25<w<1.5
1<w<1.25
0.75<w<1
0.5<w<0.75
w<0.5
ACSL1
ACAA1
APOA2
PDPK1
CPT2
ACSL4
APOA1
APOC3
GK
SLC27A1
ACSL5
CPT1A
ACADM
PCK1
DBI
PCK2
CYP27A1
ACSL3
FABP5
ACOX1
SLC27A4
SORBS1
EHHADH
FABP1
HMGCS2
SCD
SCP2
ILK
ACOX2
ME1
SLC27A2
RXRB
FABP3
ACOX3
GK
ACSL1
CYP27A1
ACOX3
APOC3
ACOX2
ACSL4
ACSL3
ME1
RXRB
SCP2
APOA1
FABP3
ILK
APOA2
EHHADH
SORBS1
SLC27A4
PCK2
SLC27A2
FABP5
ACSL5
SCD
ACAA1
SLC27A1
CPT2
FABP1
PCK1
HMGCS2
ACOX1
CPT1A
ACADM
PDPK1
DBI

Pathway: KEGG_ANTIGEN_PROCESSING_AND_PRESENTATION
There are 39 genes in this pathway

CMS3
Hub Gene (CMS3): TAP2
Weight Factor: 1.358
Hub Gene (CMS124): HLA−DRA
Weight Factor: 0.964

CMS124
Hub Gene (CMS124): HLA−DRA
Weight Factor: 1.403
Hub Gene (CMS3): TAP2
Weight Factor: 0.94

w>1.5	$1.25<w<1.5$	$1<w<1.25$	$0.75<w<1$	$0.5<w<0.75$	$w<0.5$

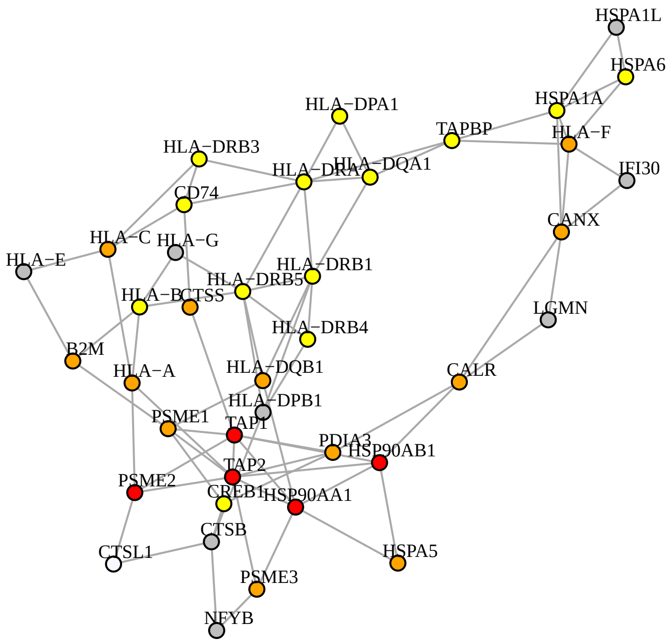

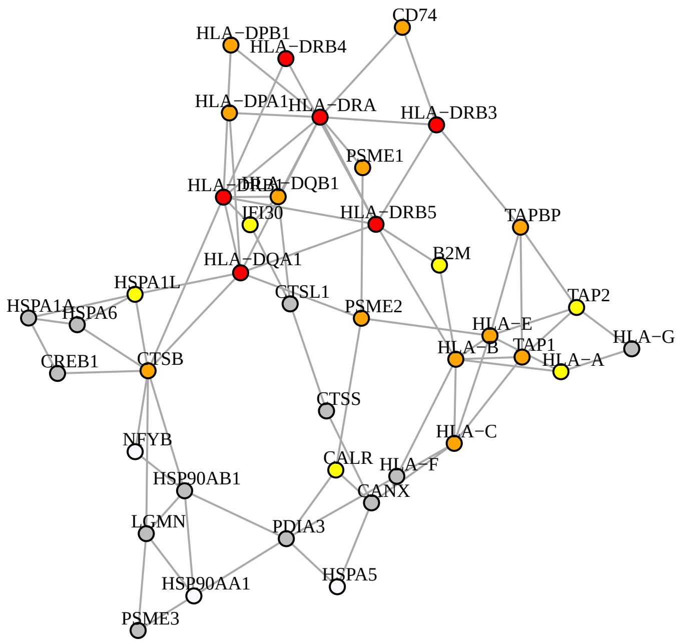

Supplement: S1 Fig — (PDF) [file pone.0221444.s001.pdf]
